# Mitochondria-dependent ferroptosis plays a pivotal role in doxorubicin cardiotoxicity

**DOI:** 10.1172/jci.insight.169756

**Published:** 2023-03-22

**Authors:** Tomonori Tadokoro, Masataka Ikeda, Tomomi Ide, Hiroko Deguchi, Soichiro Ikeda, Kosuke Okabe, Akihito Ishikita, Shouji Matsushima, Tomoko Koumura, Ken-ichi Yamada, Hirotaka Imai, Hiroyuki Tsutsui

## Abstract

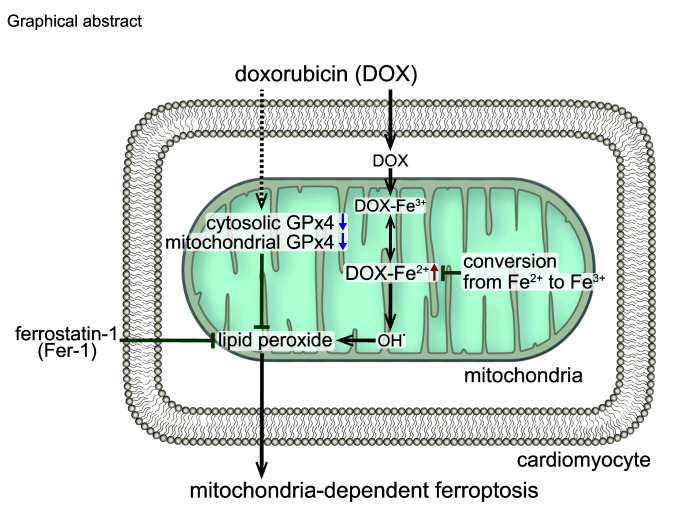

Original citation: *JCI Insight*. 2020;5(9):e132747. https://doi.org/10.1172/jci.insight.132747

Citation for this corrigendum: *JCI Insight*. 2023;8(6):e169756. https://doi.org/10.1172/jci.insight.169756

The authors recently became aware that the Mito-FerroGreen (MFG) used in this study is not an Fe^2+^ chelator, but instead reduces Fe^2+^ via conversion to Fe^3+^. As MFG mediates reduction of Fe^2+^ and suppresses ferroptosis, the overall conclusions are not affected. For clarity, the authors have removed references to MFG-mediated chelation throughout, updated the Graphical Abstract, and renumbered the reference list to refer to Hirayama et al. (1) when first describing the use of MFG in the Results section. The text and Graphical Abstract have been updated in the HTML version and PDF. The *Journal* has also published an online version of the original article with the incorrect statements crossed out and the modified text printed in red (Supplemental File, Redaction).

The authors regret the errors.

## Supplementary Material

redacted article

## Version 1 03/22/2023

Electronic publication
