# Supplementary material for: Mitochondria-dependent ferroptosis plays a pivotal role in doxorubicin cardiotoxicity
Source: JCI Insight. 2023 Mar 22;8(6):e169756. doi: 10.1172/jci.insight.169756 (PMC10070098; doi:10.1172/jci.insight.169756)
Supplement: redacted article [file jciinsight-8-169756-s224.pdf]

# Mitochondria-dependent ferroptosis plays a pivotal role in doxorubicin cardiotoxicity

**Tomonori Tadokoro,[1] Masataka Ikeda,[1] Tomomi Ide,[2] Hiroko Deguchi,[1] Soichiro Ikeda,[1] Kosuke Okabe,[1] Akihito Ishikita,[1] Shouji Matsushima,[3] Tomoko Koumura,[4] Ken-ichi Yamada,[5] Hirotaka Imai,[4] and Hiroyuki Tsutsui[1]**

[1]Department of Cardiovascular Medicine and [2]Department of Experimental and Clinical Cardiovascular Medicine, Faculty of Medical Sciences, Kyushu University, Fukuoka, Japan. [3]Department of Cardiovascular Medicine, Kyushu University Hospital, Fukuoka, Japan. [4]Department of Hygienic Chemistry and Medical Research Laboratories, School of Pharmaceutical Sciences, Kitasato University, Tokyo, Japan. [5]Physical Chemistry for Life Science Laboratory, Faculty of Pharmaceutical Sciences, Kyushu University, Fukuoka, Japan.

**Doxorubicin (DOX), a chemotherapeutic agent, induces a cardiotoxicity referred to as doxorubicin-induced cardiomyopathy (DIC). This cardiotoxicity often limits chemotherapy for malignancies and is associated with poor prognosis. However, the molecular mechanism underlying this cardiotoxicity is yet to be fully elucidated. Here, we show that DOX downregulated glutathione peroxidase 4 (GPx4) and induced excessive lipid peroxidation through DOX-$Fe^{2+}$ complex in mitochondria, leading to mitochondria-dependent ferroptosis; we also show that mitochondria-dependent ferroptosis is a major cause of DOX cardiotoxicity. In DIC mice, the left ventricular ejection fraction was significantly impaired, and fibrosis and TUNEL$^+$ cells were induced at day 14. Additionally, GPx4, an endogenous regulator of ferroptosis, was downregulated, accompanied by the accumulation of lipid peroxides, especially in mitochondria. These cardiac impairments were ameliorated in GPx4 Tg mice and exacerbated in GPx4 heterodeletion mice. In cultured cardiomyocytes, GPx4 overexpression or ~~iron chelation targeting $Fe^{2+}$~~ $Fe^{2+}$ reduction in mitochondria prevented DOX-induced ferroptosis, demonstrating that DOX triggered ferroptosis in mitochondria. Furthermore, concomitant inhibition of ferroptosis and apoptosis with ferrostatin-1 and zVAD-FMK fully prevented DOX-induced cardiomyocyte death. Our findings suggest that mitochondria-dependent ferroptosis plays a key role in progression of DIC and that ferroptosis is the major form of regulated cell death in DOX cardiotoxicity.**

## Introduction

Doxorubicin (DOX) is a chemotherapeutic agent that induces cardiotoxicity, leading to a progressive, chronic, life-threatening cardiomyopathy, referred to as DOX-induced cardiomyopathy (DIC). DIC is a lethal cardiomyopathy that is associated with a worse prognosis than other cardiomyopathies and limits the availability of DOX for malignancies due to its cardiotoxicity (1). A number of studies have reported potential molecular mechanisms underlying DIC, including transcriptional dysregulation through topoisomerase IIβ (Top2b) inhibition, $Ca^{2+}$ handling abnormalities, mitochondrial iron accumulation, and mitochondrial dysfunction (2–6). In particular, oxidative stress derived from mitochondria has been established as a potential causative mechanism. Indeed, overexpression of some antioxidative enzymes, such as manganese superoxide dismutase (Mn-SOD) and glutathione peroxidase1 (GPx1), ameliorate DIC (7, 8). However, the detailed mechanism by which oxidative stress in response to DOX causes myocardial injuries is yet to be fully elucidated, and a therapeutic strategy that prevents DIC is not established.

Ferroptosis was first described by Dixon et al. as an iron-dependent regulated cell death (RCD) that can be prevented by iron chelation (9) and is currently recognized as a form of RCD that occurs as a consequence of lethal lipid oxidation (10). Thus, ferrostatin-1 (Fer-1), used as a lipophilic radical-trapping antioxidant (RTA), can prevent ferroptosis (9, 11). Angeli et al. reported that GPx4, which is an endogenous scavenger for lipid peroxides (LPs), is a key regulator of ferroptosis (12, 13). This is supported by the evidence that most ferroptosis inducers are direct or indirect inhibitors of GPx4, and many ferroptosis inhibitors are suppressors of lipid peroxidation, such as RTAs (10, 11).

**Conflict of interest:** The authors have declared that no conflict of interest exists.

**Copyright:** © 2020, American Society for Clinical Investigation.

**Submitted:** September 5, 2019
**Accepted:** April 1, 2020
**Published:** May 7, 2020.

**Reference information:** *JCI Insight*. 2020;5(9):e132747. https://doi.org/10.1172/jci.insight.132747.

*Gpx4* is a unique gene that encodes cytosolic, mitochondrial, and nucleolar isoforms (14). Of note, the active site of GPx4 contains the rare amino acid selenocysteine (Sec), which is encoded by the UGA codon (15). Each form of GPx4 plays protective roles within each organelle, and their dysregulation is associated with disease (12, 16–21). Indeed, LP levels increased and might serve as key mediators of cardiac injuries in DIC (22, 23). We thus hypothesized that the dysregulation of ferroptosis and GPx4 under DOX treatment may be involved in LP accumulation and DIC progression. To investigate the roles of ferroptosis and GPx4 in DOX-induced cardiotoxicity, we analyzed DIC in GPx4 Tg and hetero-KO (hetKO) mice; we also studied DOX-induced cell death in cultured cardiomyocytes with adenovirus harboring GPx4 and an inhibitor against ferroptosis.

## Results

*GPx4 downregulation and increased LPs cause cardiac impairment in DIC mice*. To characterize DIC in mice, we induced DIC in mice as described previously with some modification (24): administration of DOX (6 mg/kg on days 0, 2, and 4). Impairment of left ventricular ejection fraction (LVEF) in response to DOX treatment occurred on days 7 and 14 (Figure 1, A and B). In this DIC model, weight loss, representing emaciation relevant to DOX treatment, was not observed (Figure 1C). DIC mice in this model had reduced heart weight (HW) and LV weight (Figure 1, D and E), which manifested as myocardial atrophy on day 14. Furthermore, the expression of total and mitochondrial GPx4 was significantly downregulated at day 14 following initial DOX treatment at both the mRNA (Figure 1F) and protein level (Figure 1, G and H). Acrolein and malondialdehyde (MDA), representing lipid peroxidation, increased in the myocardium of DIC mice (Figure 1, I and J). In particular, MDA in the mitochondrial fraction was significantly increased in DIC, whereas MDA in the nonmitochondrial fraction was not increased (Figure 1K), suggesting that mitochondria are responsible for an increase of MDA in response to DOX. Additionally, histological analysis showed an increase in interstitial fibrosis in DIC (Figure 1L). Because ferroptosis induced by GPx4 KO has TUNEL positivity as described previously (18), we used TUNEL staining in vivo as a potential marker of ferroptosis and observed that TUNEL$^+$ cells increased in the heart of DIC mice (Figure 1M).

*GPx4 is a critical regulator in the progression of DIC*. To investigate the roles of ferroptosis and GPx4 in DIC, we examined the DIC model in GPx4 Tg mice and GPx4 hetKO mice. GPx4 was overexpressed in the heart of Tg mice (Figure 2A). GPx4 overexpression ameliorated DOX-induced cardiac impairments, including LVEF and myocardial atrophy (Figure 2, B–E), and further reduced LPs (Figure 2, F and G), fibrosis, and TUNEL$^+$ cells (Figure 2, H and I) in DIC mice. Our fundamental analysis using flow cytometry demonstrated that the nuclei in ferroptotic cells induced by silencing GPx4 using siRNA could be stained via TUNEL staining in addition to apoptotic cells induced by staurosporine (Supplemental Figure 1, A–D; supplemental material available online with this article; https://doi.org/10.1172/jci.insight.132747DS1). Moreover, GPx4 overexpression using adenoviruses did not prevent the cleaved caspase cascade, representing apoptosis, induced by DOX in cultured cardiomyocytes (Supplemental Figure 2, A and B). Taken together, reduced TUNEL$^+$ cells in Tg mice under DOX treatment were ferroptotic, as ferroptosis was defined as a cell death regulated by GPx4 (25). In contrast, heterodeletion of GPx4 exacerbated DOX-induced cardiac impairments, including LVEF, myocardial atrophy (Figure 3, A–E), LP accumulation (Figure 3, F and G), fibrosis, and TUNEL$^+$ cells (Figure 3, H and I), in DIC mice while GPx4-heterodeletion mice showed no cardiac phenotypes without DOX treatment. Given that ferroptosis is defined as a cell death regulated by GPx4 (25), these results suggest that GPx4 is a critical regulator of DIC progression and that ferroptosis plays a key role in the progression of DIC.

*Ferroptosis is involved in DOX-induced cell death in cultured cardiomyocytes*. To further investigate the role of ferroptosis in DIC, cultured cardiomyocytes, along with nonmyocytes (including cardiac fibroblasts and cardiac endothelial cells) treated with DOX were examined. Since MDA, mitochondrial LPs labeled with MitoPeDPP, and cell death were induced in a DOX-concentration dependent manner in cultured cardiomyocytes (Supplemental Figure 3, A–C), we treated cardiac cells with DOX at 2 μM for 30 hours. GPx4 was downregulated in response to DOX in cultured cardiomyocytes (Figure 4, A and B) but not in nonmyocytes (Supplemental Figure 4A). There was an increase in MDA in both cultured cardiomyocytes and nonmyocytes from whole cell lysates (Figure 4C and Supplemental Figure 4B). Further analysis revealed that MDA in the mitochondrial fraction was markedly increased, whereas MDA in the nonmitochondrial fraction was not increased in cultured cardiomyocytes (Figure 4C). These results suggest that lipid peroxidation was induced in the mitochondria after DOX treatment. In addition, the levels of mitochondrial LPs labeled with MitoPeDPP were also significantly increased

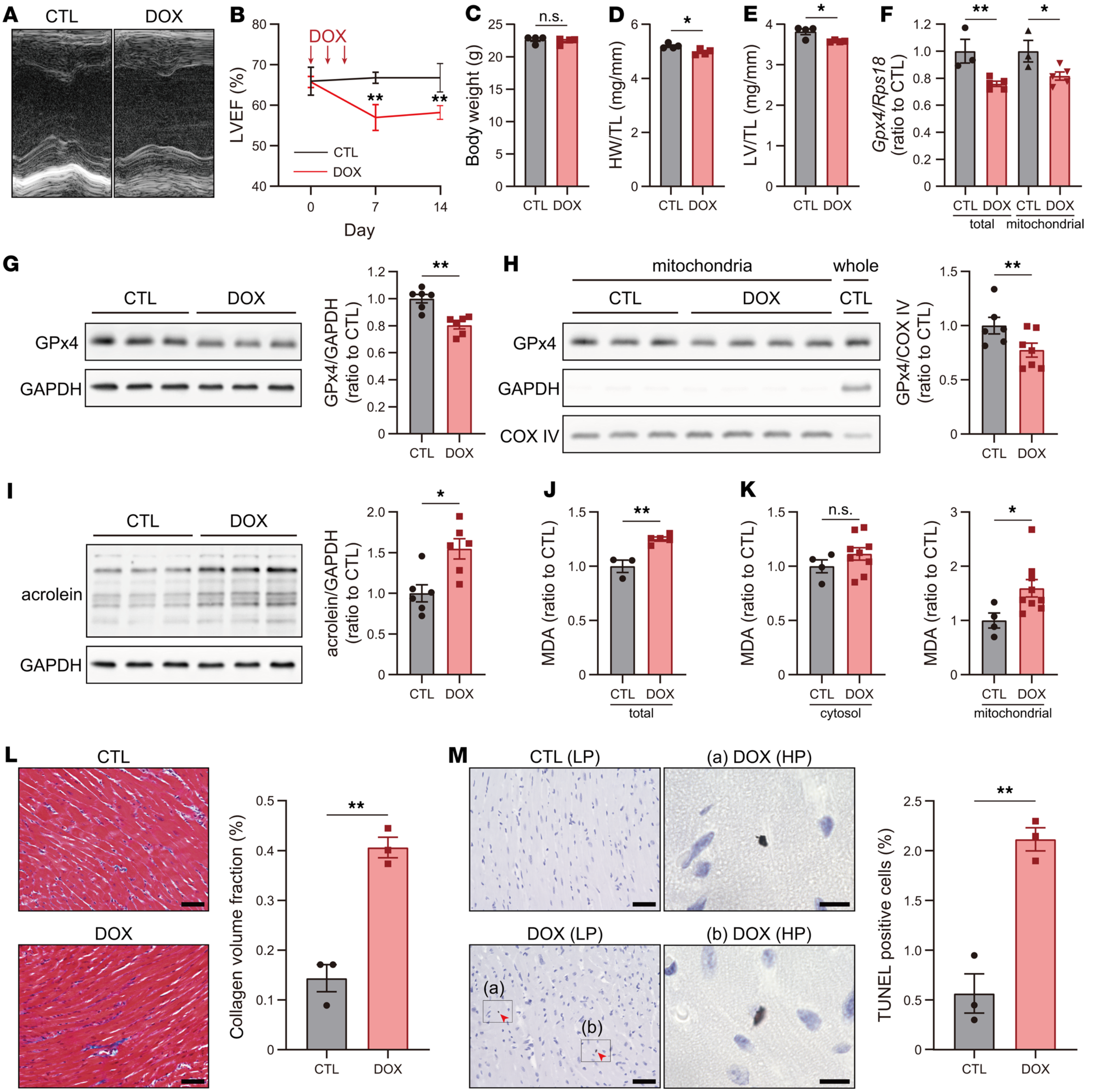


**Figure 1. GPx4 is downregulated and LP levels increase in the myocardium of DIC mice.** (**A**) Echocardiographic images of CTL mice or DIC mice at day14. (**B**) LVEF, left ventricular ejection fraction (*n* = 4). (**C**) Body weight at day 14 (*n* = 4). (**D**) Heart weight (HW), normalized by tibial length (TL) at day 14 (*n* = 4). (**E**) Left ventricle (LV) weight, normalized by TL at day 14 (*n* = 4). (**F**) Total (left) and mitochondrial (right) *Gpx4* expression in the myocardium at day 14 was quantified by real-time PCR (*n* = 3 and 5, respectively). (**G**) Western blot of GPx4 from heart tissue lysates at day 14 (*n* = 6, each). (**H**) Western blot of mitochondrial lysates obtained from the heart at day 14, for GPx4 (*n* = 6–7). (**I**) Western blot of acrolein in heart tissue lysates at day 14 (*n* = 6). (**J**) Malondialdehyde (MDA) levels in the myocardium at day 14 were measured by thiobarbituric acid reactive substances (TBARs) assay (*n* = 3 and 5). (**K**) MDA levels in the cytosolic fraction (left) and mitochondrial fraction (right) of the myocardium (*n* = 4 and 9, respectively). (**L**) Interstitial fibrosis in the LV and Masson trichrome staining in CTL and DIC mice. Scale bars: 50 μm (*n* = 3). (**M**) TUNEL staining in CTL and DIC mice at the low power (LP) (scale bar: 50 μm) and the high power (HP) (scale bar: 10 μm), and quantification of the number of TUNEL⁺ cells (*n* = 3). Arrowheads indicate TUNEL⁺ nuclei. Data are shown as the mean ± SEM. Statistical significance was determined using 2-tailed Student's *t* test. *$P < 0.05$, **$P < 0.01$.

after DOX treatment in both cardiomyocytes and nonmyocytes (Figure 4, D and E, and Supplemental Figure 4C). Cell death was induced by DOX treatment in both cardiomyocytes and nonmyocytes (Supplemental Figure 3C and Supplemental Figure 4D). Fer-1, an RTA-type ferroptosis inhibitor, significantly decreased LP levels (Figure 4, E and F) and prevented the DOX-induced cell death in cardiomyocytes (Figure 4G), whereas Fer-1 did not

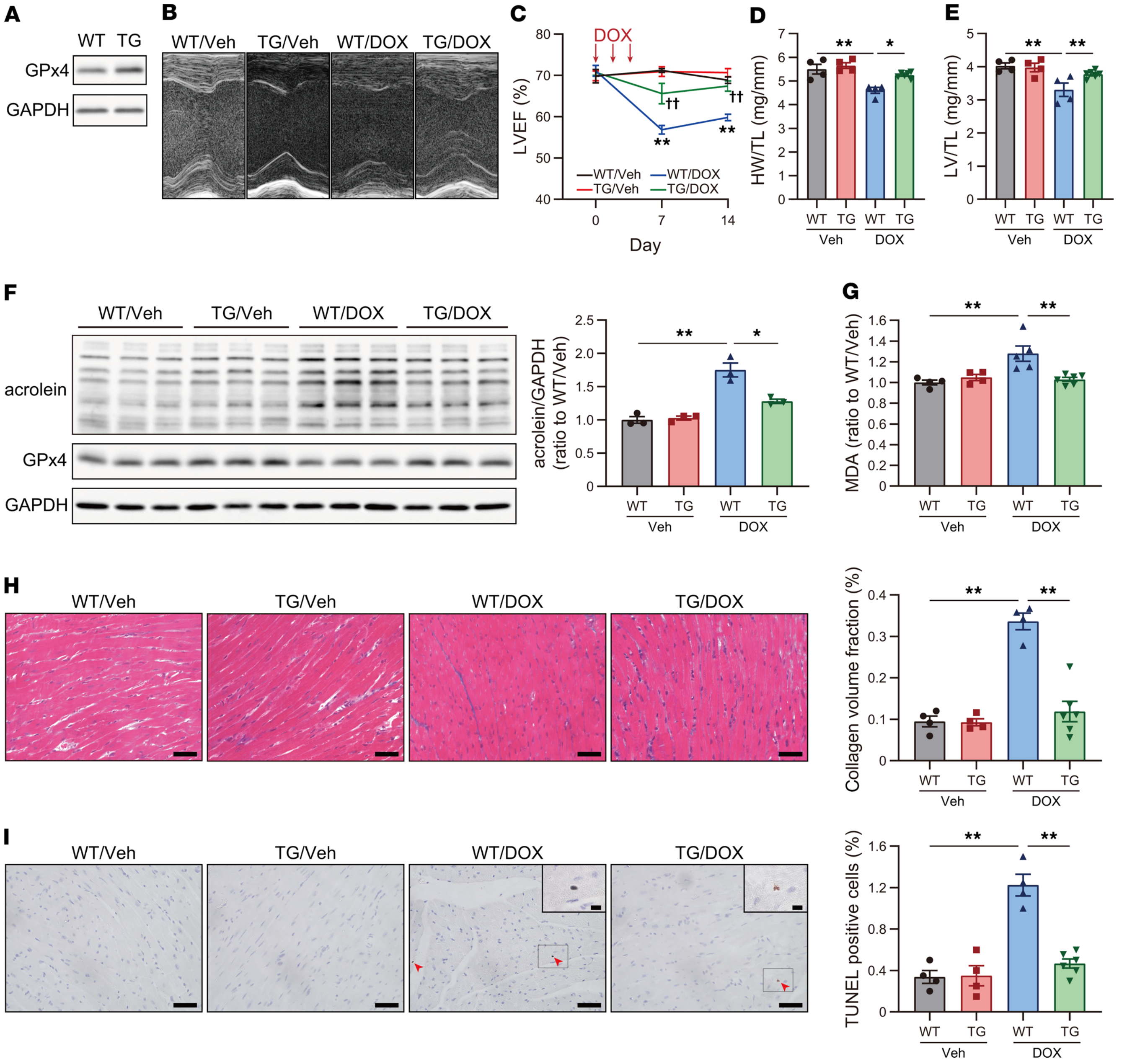


**Figure 2. GPx4 overexpression ameliorates DIC.** (**A**) Western blot of GPx4 in heart tissue lysates from WT and GPx4 Tg mice. (**B**) Echocardiographic images of WT and GPx4 Tg mice, treated with vehicle (Veh) or doxorubicin (DOX), at day 14. (**C**) LVEF ($n$ = 4–6). **$P < 0.01$ vs. WT/Veh. ††$P < 0.01$ vs. WT/DOX. Statistical significances at day 7 and 14 were determined using 1-way ANOVA with a post hoc Tukey's HSD test. (**D**) Heart weight, normalized by tibial length (TL) at day 14 ($n$ = 4–6). (**E**) Left ventricle (LV) weight, normalized by TL at day 14 ($n$ = 4–6). (**F**) Western blot of acrolein and GPx4 in heart tissue lysates at day 14 ($n$ = 3, each group). (**G**) Malondialdehyde (MDA) in the myocardium at day 14 was measured by thiobarbituric acid reactive substances (TBARs) assay ($n$ = 4–6). (**H**) Interstitial fibrosis in the LV evaluated using Masson trichrome staining in WT and Tg mice treated with Veh or DOX. Scale bar: 50 μm ($n$ = 4–6). Quantification of interstitial fibrosis, assessed by collagen volume fraction (%, interstitial fibrosis per total myocardium) (right panel). (**I**) TUNEL staining in WT and Tg mice treated with Veh or DOX. Scale bar: 50 μm ($n$ = 4–6). Arrowheads indicate TUNEL$^+$ nuclei. Representative image of TUNEL$^+$ cells at a high power (upper right panel). Scale bar: 10 μm ($n$ = 4–6). Data are shown as the mean ± SEM. Statistical significance was determined using 1-way ANOVA with a post hoc Tukey's HSD test. *$P < 0.05$, **$P < 0.01$.

suppress LPs, and prevent cell death induced by DOX in nonmyocytes, and even aggravated it at higher dose of Fer-1 (Supplemental Figure 4, B–D). These results suggest that GPx4 downregulation and ferroptosis are induced in cardiomyocytes — but not in nonmyocytes — after DOX treatment and that lipid peroxidation in the mitochondrial membrane probably triggers DOX-induced ferroptosis in cardiomyocytes.

*DOX-induced ferroptosis is triggered in mitochondria*. As shown earlier, LPs were significantly increased in mitochondria, but not in other organelles, in response to DOX. To further understand the roles of

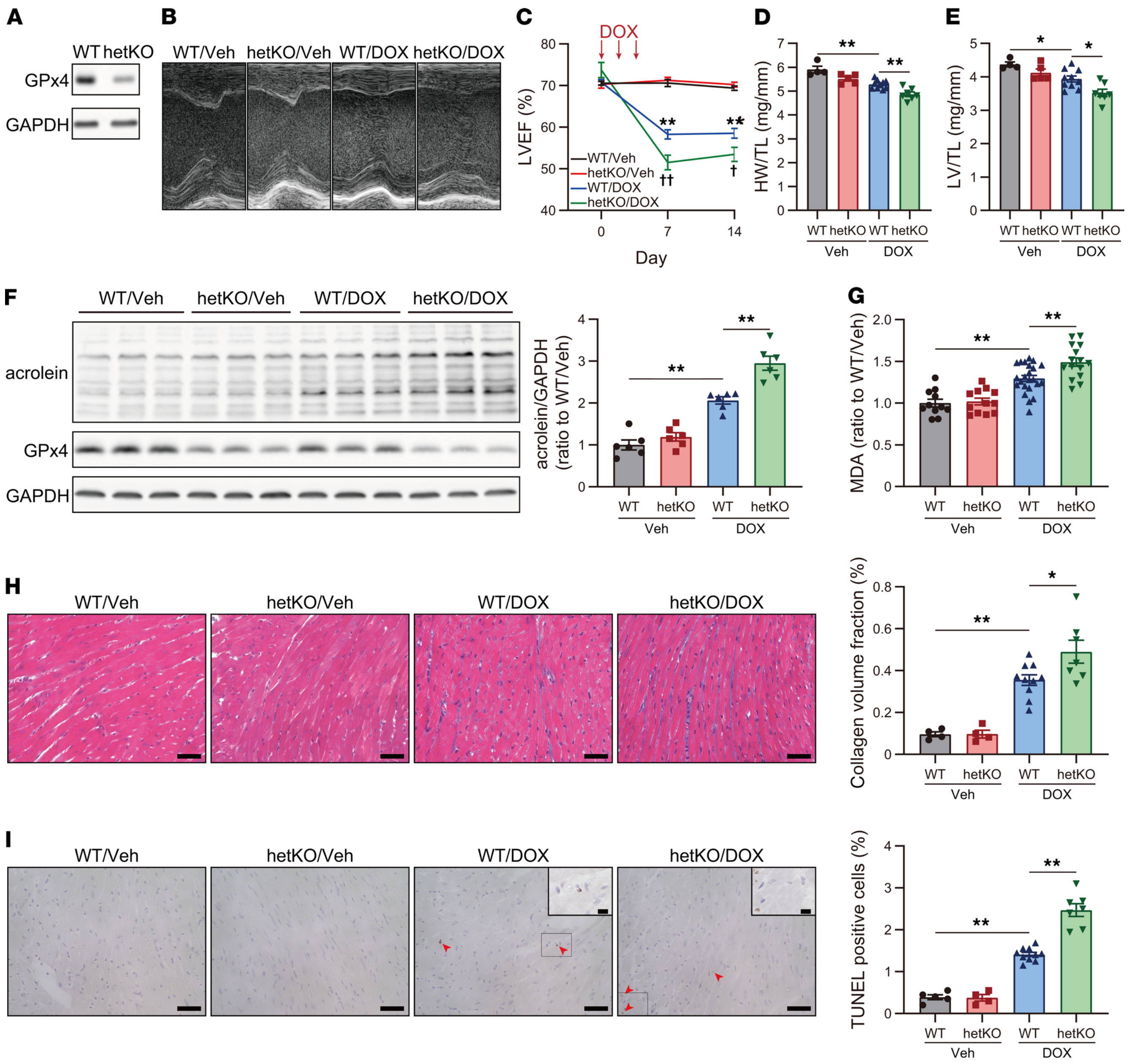


**Figure 3. Heterodeletion of GPx4 aggravates DIC.** (**A**) Western blot of GPx4 in heart tissue lysates from WT and GPx4 hetero-KO (hetKO) mice. (**B**) Echocardiographic images of WT and hetKO mice, treated with vehicle (Veh) and DOX, at day 14. (**C**) LVEF ($n$ = 4–10). $^{**}P < 0.01$ vs. WT/Veh. †$P < 0.01$, ††$P < 0.01$ vs. WT/DOX. Statistical significances at day 7 and 14 were determined using 1-way ANOVA with a post hoc Tukey's HSD test. (**D**) Heart weight, normalized by tibial length (TL) at day 14 ($n$ = 4–10). (**E**) Left ventricle (LV) weight, normalized by TL at day 14 ($n$ = 4–10). (**F**) Western blot of acrolein and GPx4 in heart tissue lysates at day 14 ($n$ = 6, each). (**G**) Malondialdehyde (MDA) in the myocardium at day 14 was measured by thiobarbituric acid reactive substances (TBARs) assay ($n$ = 11–23). (**H**) Interstitial fibrosis in the LV evaluated using Masson trichrome staining in WT and hetKO mice treated with Veh or DOX. Scale bar: 50 μm ($n$ = 4–10). Quantification of interstitial fibrosis, assessed by collagen volume fraction (%, interstitial fibrosis per total myocardium) (right panel). (**I**) TUNEL staining in WT and hetKO mice treated with Veh or DOX. Scale bar: 50 μm (n = 4–10). Arrowheads indicate TUNEL$^+$ nuclei. Representative image of TUNEL$^+$ cells at a high power (upper right panel). Scale bar: 10 μm ($n$ = 4–10). Data are shown as the mean ± SEM. Statistical significance was determined using 1-way ANOVA with a post hoc Tukey's HSD test. $^*P < 0.05$, $^{**}P < 0.01$.

GPx4 and mitochondria in DOX-induced ferroptosis in cultured cardiomyocytes, we cloned cDNAs of rat*Gpx4* with or without a mitochondrial targeting signal (MTS) and produced 2 kinds of adenoviruses harboring nonmitochondrial (cytosolic) and mitochondrial *Gpx4* (Supplemental Figure, 5 and 6). These adenoviruses overexpressed each form of cytosolic GPx4 or mitochondrial GPx4 in a multiplicity of infection–dependent (MOI-dependent) manner (Figure 5A). Cultured cardiomyocytes were infected with adenoviruses (3 MOI, 48 hours). Unexpectedly, infection of adenovirus harboring cytosolic *Gpx4*

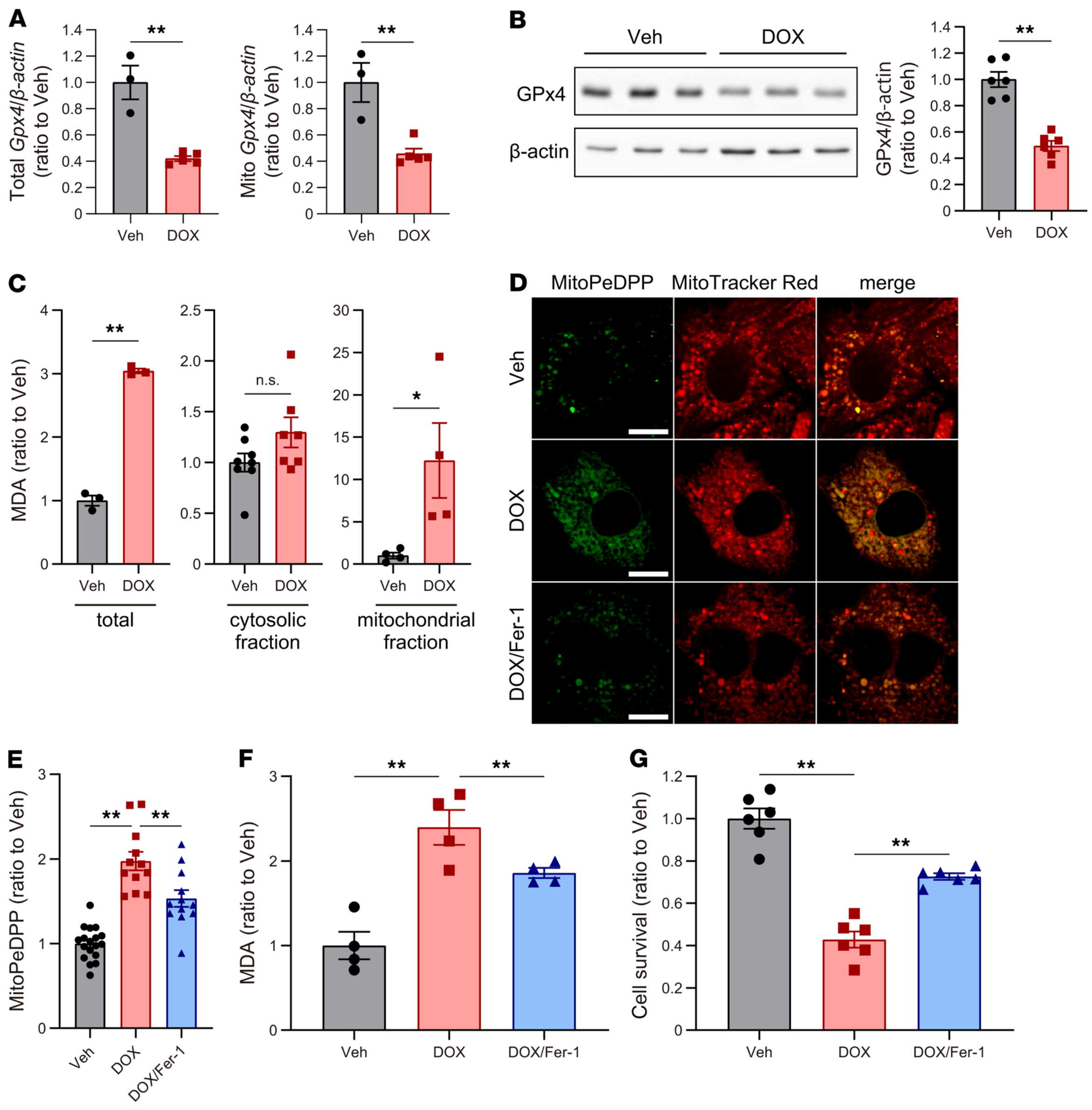


**Figure 4. Ferroptosis is involved in DOX-induced cell death in cultured cardiomyocytes.** (**A**) Total (left) and mitochondrial *Gpx4* (right) expression in the cultured cardiomyocytes treated with vehicle or doxorubicin (DOX) was quantified by real-time PCR ($n$ = 3 and 5, respectively). (**B**) Western blot of GPx4 in lysates from the cultured cardiomyocytes ($n$ = 6). (**C**) MDA in the cultured cardiomyocyte was measured using the TBARs assay (left; $n$ = 3). MDA in the cytosolic fraction of the cultured cardiomyocytes (middle; $n$ = 8 and 7, each). MDA in the mitochondrial fraction of the cultured cardiomyocytes (right; $n$ = 4). (**D**) Representative fluorescence imaging of mitochondrial LPs using MitoPeDPP in cultured cardiomyocytes under control conditions, in the presence of DOX (2 μM, 30 hours) or Fer-1 (50 μM, 30 hours) (green, left panels). Mitochondria were counterstained with MitoTracker Red (red, central panels). Scale bars: 10 μm. (**E**) Mitochondrial lipid peroxidation was measured using MitoPeDPP ($n$ = 12–18). (**F**) MDA in the cultured cardiomyocyte, treated with DOX or Fer-1, was measured using the TBARs assay ($n$ = 4, each). (**G**) Cell viability was assessed 30 hours after treatment with DOX or Fer-1 ($n$ = 6). Data are shown as the mean ± SEM. Statistical significance was determined using 2-tailed Student's t test (**A–C**) or 1-way ANOVA with a post hoc Tukey's HSD test (**E–G**) *$P$ < 0.05, **$P$ < 0.01.

(Ad-cytoGPx4-FLAG) increased GPx4-FLAG in both the cytosolic and mitochondrial fractions, whereas infection with adenovirus harboring mitochondrial *Gpx4* (Ad-mitoGPx4-FLAG) mainly increased GPx4 in the mitochondria (Figure 5, B and C). Using immunohistochemical electron microscopy (IHEM), we further examined the localization of GPx4 in cardiomyocytes and found that exogenous cytoGPx4-FLAG protein was distributed not only in cytosol, but also in the outer membrane and intermembrane space of mitochondria (Figure 5D, left). However, mitoGPx4-FLAG was mainly localized in mitochondria (Figure 5D, right). These results from IHEM are consistent with those from Western blotting (Figure 5B), and indicate the following: (a) a part of cytosolic GPx4 without an MTS could be transported into mitochondria and (b) mitochondrial GPx4 is mainly localized in mitochondria, while

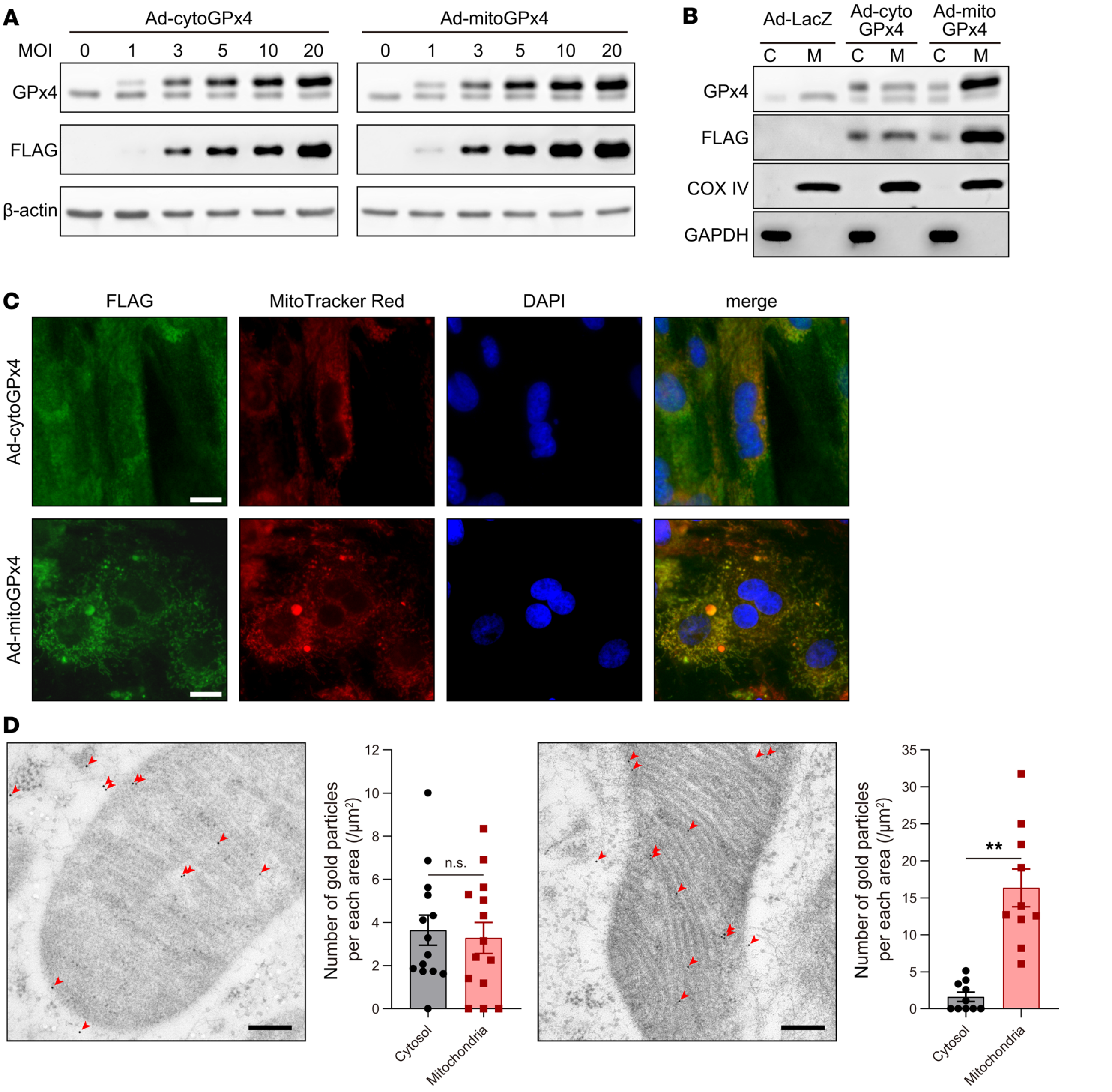


**Figure 5. Characterization of adenoviruses harboring cytosolic *Gpx4* (Ad-cytoGPx4) and mitochondrial *Gpx4* (Ad-mitoGPx4).** (**A**) GPx4 and GPx4-FLAG expression 48 hours after infection with adenoviruses harboring cytosolic and mitochondrial *Gpx4* (0, 1, 3, 5, 10, and 20 MOI). (**B**) Western blot of GPx4, GPx4-FLAG, COX IV, and GAPDH in the cytosolic (C) and mitochondrial (M) fractions. (**C**) Immunofluorescence microscopy for localization of FLAG-tagged GPx4, MitoTracker Red, and DAPI in cultured cardiomyocytes. Scale bars: 10 μm. (**D**) Immunohistochemical electron microscopy of cultured cardiomyocytes 48 hours after infection with adenoviruses (3 MOI) harboring cytosolic (left panel) and mitochondrial *Gpx4* (right). Single red arrowheads indicate gold particle. Scale bar: 200 nm. ($n$ = 14 and 10, respectively). Data are shown as the mean ± SEM. Statistical significance was determined using 2-tailed Student's $t$ test. ** $P < 0.01$.

a very small amount of mitochondrial GPx4 can be found in the cytosol; this may just be a snapshot of GPx4, as it is transported from cytosol to mitochondria. Using these adenoviruses, we investigated the role of GPx4 in cytosol and mitochondria in DOX-induced ferroptosis. While both Ad-cytoGPx4-FLAG and Ad-mitoGPx4-FLAG had no suppressive effect on DOX-induced caspase cleavages representing apoptosis (Figure 6A), they equally suppressed DOX-induced LP accumulation (Figure 6, B and C) and protected against DOX-induced cell death (Figure 6D). As mitochondrial GPx4 is mainly located at mitochondria, these results suggest that GPx4 in mitochondria plays a key role in the protection from DOX-induced LPs and ferroptosis and that DOX-induced ferroptosis is triggered in mitochondria.

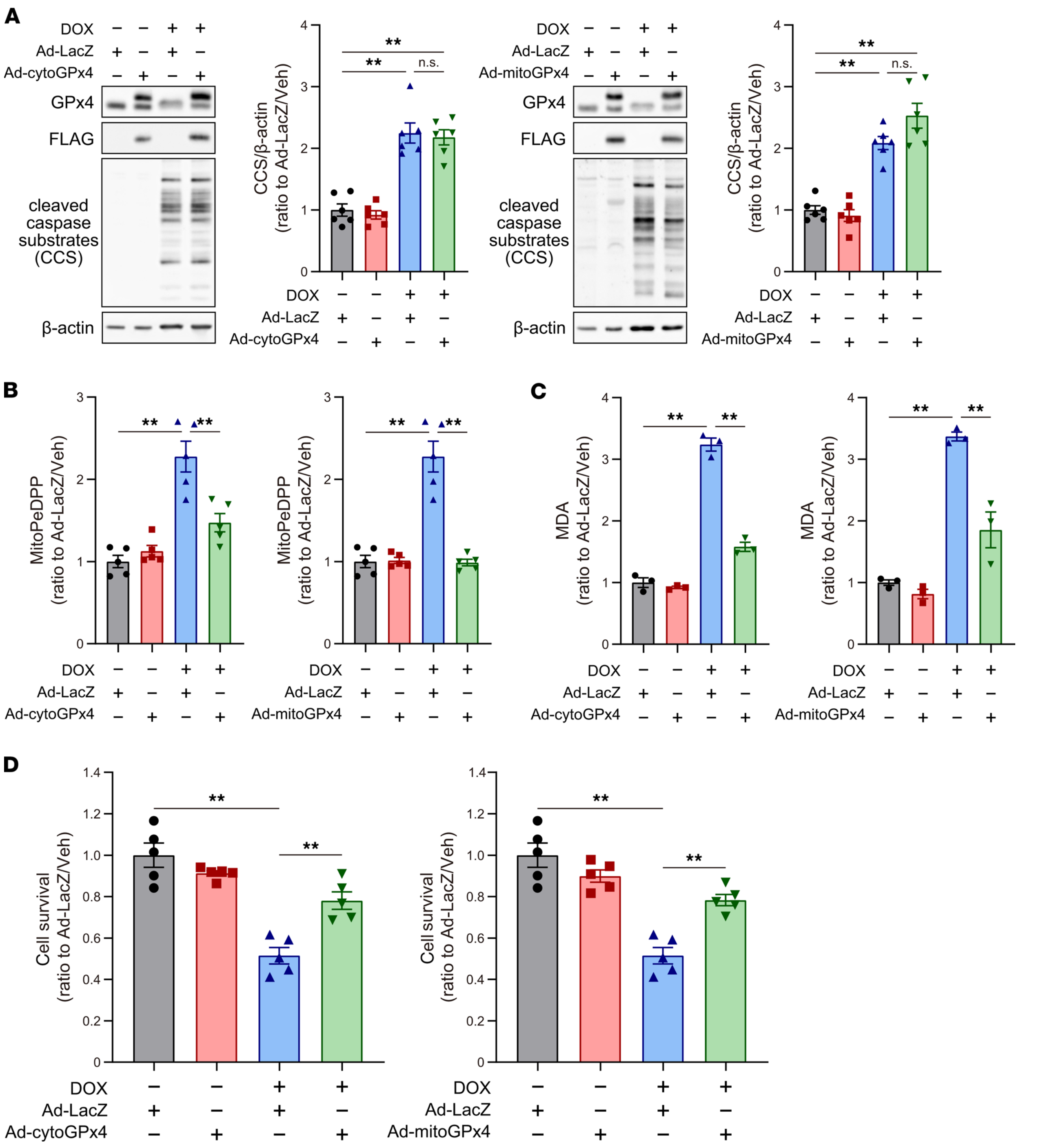


**Figure 6. The roles of cytosolic and mitochondrial GPx4 in DOX-induced ferroptosis.** (**A**) Western blot of GPx4, GPx4-FLAG, and cleaved caspase substrates in cardiomyocyte cell lysates, infected with Ad-cytoGPx4 and Ad-mitoGPx4 ($n = 6$). (**B**) Mitochondrial lipid peroxidation was measured using MitoPeDPP in the cultured cardiomyocyte, infected with Ad-cytoGPx4 and Ad-mitoGPx4 ($n = 5$). (**C**) Malondialdehyde (MDA) in the cultured cardiomyocyte, infected with Ad-cytoGPx4 and Ad-mitoGPx4, was measured using the thiobarbituric acid reactive substances (TBARs) assay ($n = 3$). (**D**) Cell viability was assessed 30 hours after treatment with DOX (2 μM; $n = 5$). Data are shown as the mean ± SEM. Statistical significance was determined using 1-way ANOVA with a post hoc Tukey's HSD test. $*P < 0.05$, $**P < 0.01$.

~~*Chelation against ferrous iron ($Fe^{2+}$), but not ferric iron ($Fe^{3+}$), in mitochondria prevents DOX-induced LPs and ferroptosis*~~ *Reduction of ferrous iron ($Fe^{2+}$), but not ferric iron ($Fe^{3+}$), in mitochondria prevents DOX-induced LPs and ferroptosis*. The source of LPs that cause ferroptosis is generally thought to be iron (ferrous iron; $Fe^{2+}$) through the Fenton reaction, and the iron chelator, deferoxamine (DFO), can suppress LPs and ferroptosis induced by RSL3 and erastin in a Ras-mutated cell line (9, 10, 12). In addition, Ichikawa et al. demonstrated that mitochondrial

non-heme iron is accumulated in response to DOX and that the iron chelators, DFO and dexrazoxane (DXZ), ameliorated DOX-induced cardiac impairments in DIC (5). In fact, we also observed an increase in non-heme iron in mitochondria, not in whole heart, in our DIC model (Supplemental Figure 7, A and B). We therefore examined the iron in mitochondria and found that iron accumulated in mitochondria in response to DOX in cultured cardiomyocytes (Figure 7, A and B). In addition, DFO and DXZ, which are chelators for $Fe^{3+}$, significantly reduced the content of iron in mitochondria under DOX treatment (Figure 7C). However, DOX-induced LP accumulation and cell death were not prevented by DFO and DXZ (Figure 7, D–F).

Considering the role of iron as a source of LPs through the Fenton reaction, the dynamics of iron under DOX treatment might be different from that in physiological conditions because DOX itself is a chelating agent for $Fe^{3+}$ and forms a DOX-$Fe^{3+}$ complex (26). DOX-$Fe^{3+}$ is reduced to DOX-$Fe^{2+}$ complex in an oxygen concentration–dependent manner (27), and then it produces hydroxyl radical ($OH^{-}$), resulting in the production of LPs (26, 28), suggesting that DOX-$Fe^{2+}$, but not free $Fe^{2+}$, plays a central role in the DOX-related LP production. Furthermore, DOX can directly extract $Fe^{3+}$ from ferritin (29), suggesting that DOX forms DOX-$Fe^{3+}$ complex without free $Fe^{3+}$ and that free $Fe^{2+}$ and $Fe^{3+}$ might not be essential for the formation of DOX-Fe complex and LP production under DOX treatment. Indeed, it was previously reported that chelation against $Fe^{2+}$ using bathophenanthroline (BPS) effectively prevented DOX-induced LPs through the inhibition of DOX-$Fe^{2+}$ (30). We thus hypothesized that ~~chelation against $Fe^{2+}$~~ conversion from $Fe^{2+}$ to $Fe^{3+}$ could prevent DOX-induced LPs and ferroptosis. ~~To investigate the role of chelating agents against Fe2+ in DOX-induced ferroptosis~~To test this hypothesis, we used Mito-FerroGreen (MFG), a mitochondria-specific $Fe^{2+}$ fluorescence indicator, as ~~a chelating agent against $Fe^{2+}$ in mitochondria~~ a converter from $Fe^{2+}$ to $Fe^{3+}$ in mitochondria through its reaction (31). This examination revealed that MFG treatment significantly prevented DOX-induced LPs and cell death (Figure 7, G–I), indicating that DOX-$Fe^{2+}$ produced LPs in mitochondria and induced mitochondria-dependent ferroptosis. These results suggest that DOX-$Fe^{2+}$ plays a central role in DOX-related Fenton reaction and that ~~chelators against $Fe^{2+}$~~ $Fe^{2+}$ reduction, but not $Fe^{3+}$, in mitochondria effectively prevent DOX-induced LPs and mitochondria-dependent ferroptosis.

*Ferroptosis is the major form of RCDs in cardiomyocytes after DOX treatment*. Inhibition of ferroptosis by Fer-1, Ad-cytoGPx4, or Ad-mitoGPx4 failed to completely abrogate DOX-induced cell death. On the other hand, cleavage of caspases was significantly increased in DOX-treated cardiomyocytes (Figure 6A and Supplemental Figure 2, A and B), suggesting that apoptosis is also an important form of RCD in DOX-induced cell death. In addition, Zhang et al. reported that necroptosis is also another form of RCD in DOX-induced cell death (4). However, an inhibition of RIP1K using necrostatin-1 or the knockdown of *Ripk3* had no significant protection against DOX-induced cell death (Supplemental Figure 8, A and B), and *Ripk3* knockdown suppressed DOX-induced caspase cleavage (Supplemental Figure 8C), suggesting that necroptosis through RIP3K is a relatively minor form of RCD under DOX treatment and might be recognized as a part of apoptosis. These results suggest that apoptosis and ferroptosis are major RCDs induced by DOX. To clarify the independence of ferroptosis from apoptosis, we examined the effects of combining Fer-1 with zVAD-FMK (zVAD), an apoptosis inhibitor, on DOX-induced cell death at 30 hours after DOX treatment. zVAD treatment completely suppressed cleavage of caspases and caspase activity induced by DOX, whereas Fer-1 did not (Figure 8, A and B). Furthermore, Fer-1 did not reduce cytochrome c in the cytosol, which represents mitochondria-dependent apoptosis, under DOX treatment (Figure 8C), and the number of apoptotic cells, labeled by annexin V binding (Figure 8D). On the other hand, Fer-1 significantly suppressed mitochondrial LP in response to DOX, while zVAD did not (Figure 8E). These results suggest that ferroptosis is induced independently of apoptosis during DOX-induced cell death.

To investigate the significance of ferroptosis in DOX-induced cardiotoxicity in contrast to apoptosis, we further examined the time-course of GPx4 and cleaved caspases after DOX treatment, and we found that cleaved caspases reach a peak 10 hours after DOX treatment, whereas GPx4 was gradually downregulated until 30 hours after DOX treatment (Figure 9A). We examined the effect of Fer-1 and/or zVAD on DOX-induced cell death at each time point and found that zVAD or Fer-1 partially prevented DOX-induced cell death, while the concomitant inhibition of ferroptosis and apoptosis with Fer-1 and zVAD fully prevented DOX-induced cell death at any time point (Figure 9B and Supplemental Figure 9). These results suggested that ferroptosis and apoptosis cooperatively work to induce DOX-induced cell death. From the data shown in Figure 9B, the mechanisms underlying DOX-induced cell death are summarized in Figure 9C, suggesting that ferroptosis, along with apoptosis, is the major form of RCD in DOX-induced cell death in cardiomyocytes at any phase after DOX treatment.

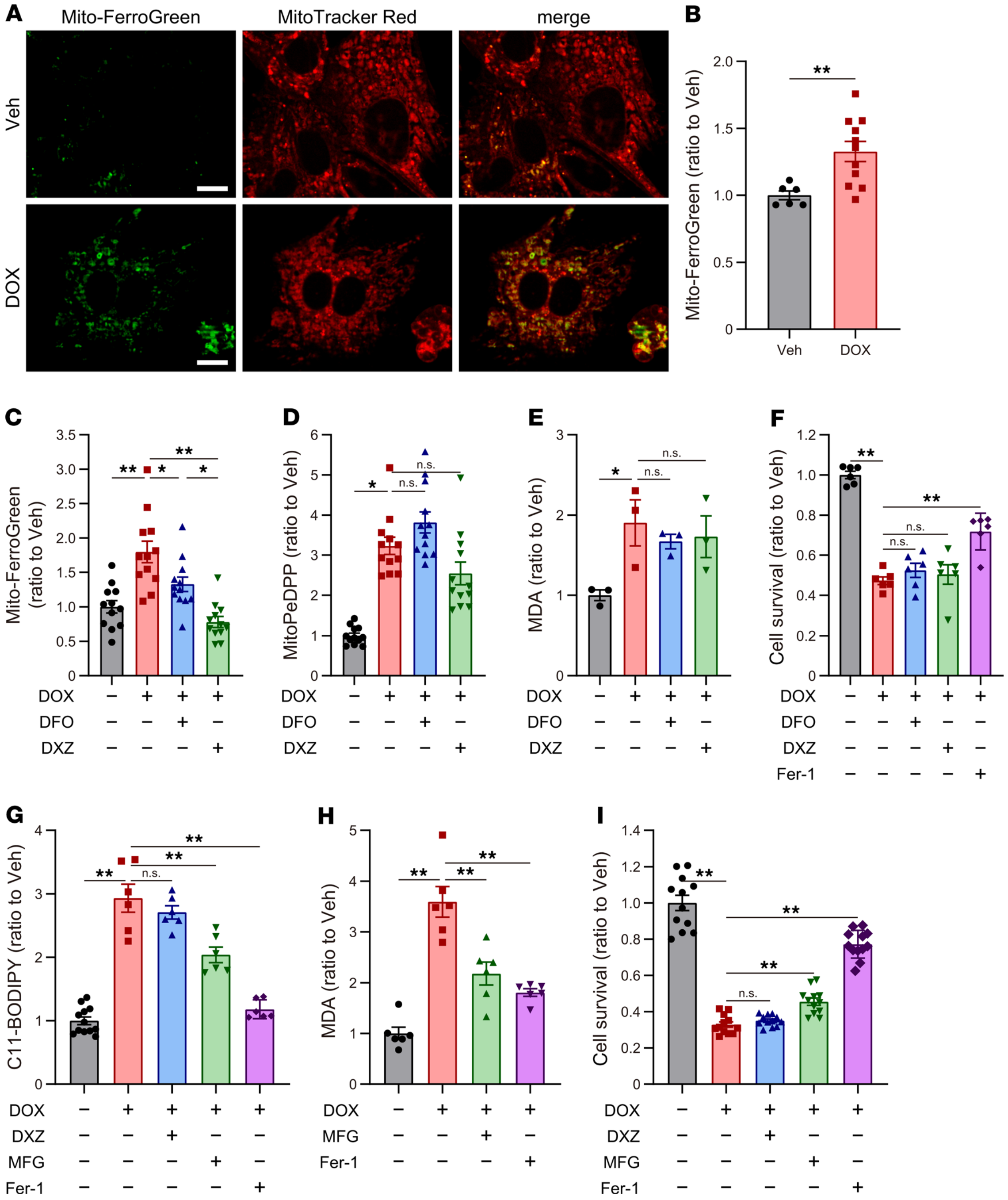


**Figure 7. The role of iron in DOX-induced ferroptosis.** (**A**) Representative fluorescence imaging of mitochondrial iron using Mito-FerroGreen (MFG) in cultured cardiomyocytes, treated with vehicle (Veh) and doxorubicin (DOX 2 μM, 30 hours) (green, left panels). The mitochondria of the cells were counterstained with MitoTracker Red (red, central panels). Scale bars: 10 μm. (**B**) Quantification of relative intensity of MFG in cultured cardiomyocytes ($n$ = 6 and 11). (**C**) Non-heme iron in the mitochondria of cultured cardiomyocytes after treatment with DOX, deferoxamine (DFO, 100 μM), and dexrazoxane (DXZ, 1000 μM), assessed using MFG ($n$ = 12). (**D**) Mitochondrial lipid peroxidation was measured using MitoPeDPP ($n$ = 12). (**E**) Malondialdehyde (MDA) in the cultured cardiomyocyte was measured using the thiobarbituric acid reactive substances (TBARs) assay ($n$ = 3). (**F**) Cell viability was assessed 30 hours after treatment with DOX (2 μM; $n$ = 12). (**G**) Lipid peroxidation was measured using C11-BODIPY 581/591 ($n$ = 6–12) in the cultured cardiomyocytes 30 hours after DOX treatment (2 μM), with DXZ (1000 μM), MFG (10 μM), and Fer-1 (50 μM). (**H**) Malondialdehyde (MDA) in the cultured cardiomyocytes, treated with DOX, MFG, and Fer-1 ($n$ = 6, each). (**I**) Cell viability was assessed 30 hours after DOX treatment (2 μM) with DXZ (1000 μM), MFG (10 μM), and Fer-1 (50 μM) ($n$ = 12, each). Data are shown as the mean ± SEM. Statistical significance was determined using 2-tailed Student's t test (**B**) or 1-way ANOVA with a post hoc Tukey's HSD test (**C–I**). *$P$ < 0.05, **$P$ < 0.01.

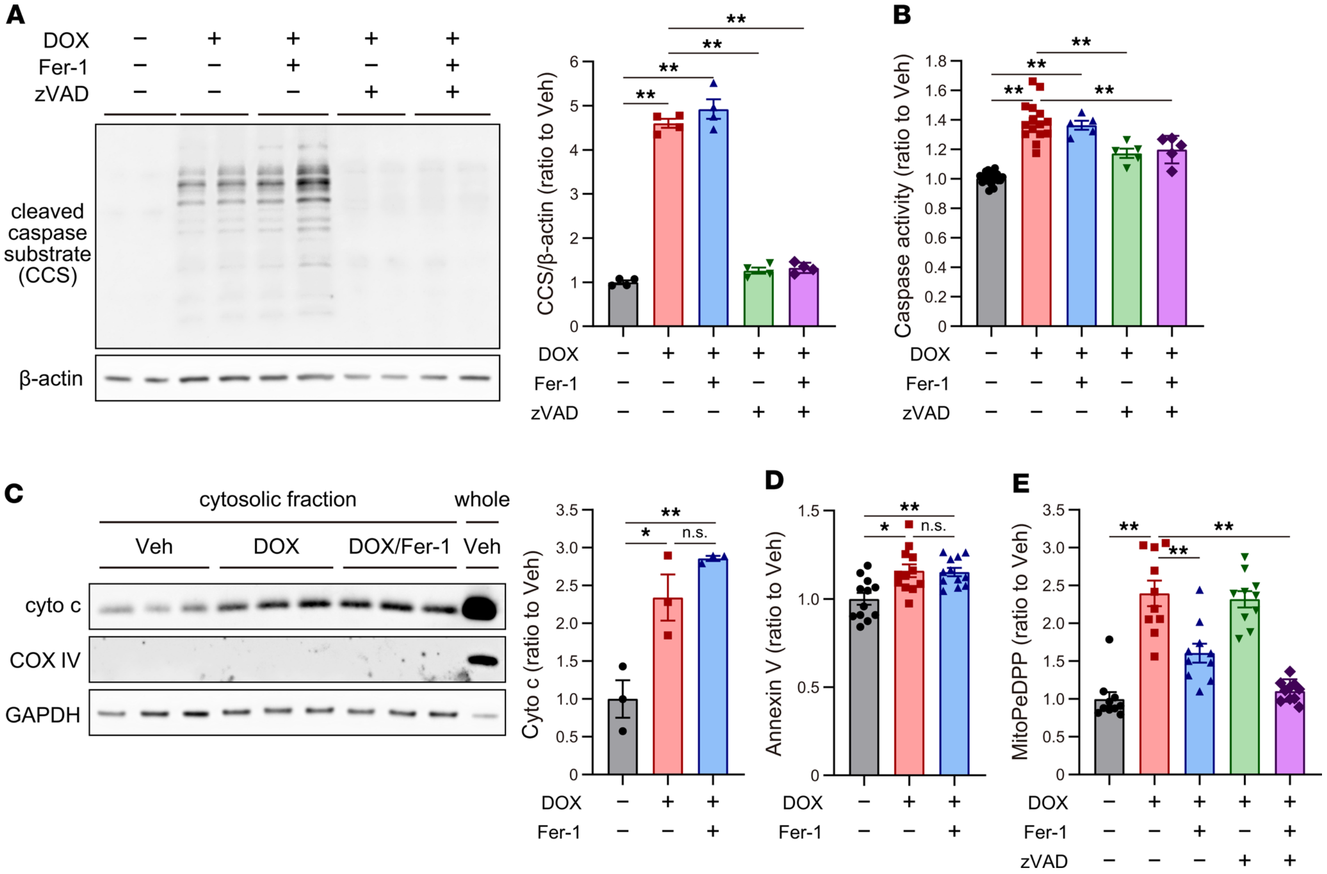


**Figure 8. Independence of ferroptosis from apoptosis under DOX treatment.** (**A**) Western blot of cleaved caspase substrates in cardiomyocyte cell lysates at 30 hours after treatment ($n$ = 4). (**B**) Caspase activity was measured using CellEvent Caspase-3/7 Green Detection Reagent at 30 hours after treatment ($n$ = 5–20). (**C**) Cytosolic cytochrome c (cyto c), representing mitochondria-dependent apoptosis, in cultured cardiomyocytes, treated with vehicle (Veh) and doxorubicin (DOX), and DOX/ferrostatin-1 (Fer-1), at 30 hours after treatment ($n$ = 3). (**D**) Annexin V binding was measured using annexin V, Alexa Fluor 488 conjugate ($n$ = 12). (**E**) Mitochondrial lipid peroxidation was measured using MitoPeDPP at 30 hours after treatment ($n$ = 10). Data are shown as the mean ± SEM. Statistical significance was determined using 1-way ANOVA with a post hoc Tukey's HSD test. *$P$ < 0.05, **$P$ < 0.01.

## Discussion

Ferroptosis is a newly described form of RCD that can be prevented by an iron chelator, and GPx4 is recognized as an important endogenous regulator of ferroptosis (9, 12). Recently, Stockwell et al. described that ferroptosis is a form of RCD that occurs as a consequence of lethal lipid peroxidation and that the criteria for ferroptosis are variable and dependent on the mechanism via which ferroptosis is triggered (10). Our primary findings are as follows: (a) GPx4 was downregulated in response to DOX treatment; (b) GPx4 overexpression prevented the progression of DIC and GPx4 heterodeletion exacerbated it; (c) DOX-induced ferroptosis is triggered in mitochondria; (d) ~~iron chelator against $Fe^{2+}$~~$Fe^{2+}$ reduction, but not $Fe^{3+}$, in mitochondria effectively prevented DOX-induced ferroptosis; and (e) ferroptosis is the major form of RCD, along with apoptosis, under DOX treatment.

Myocardial injuries induced by DOX can be classified into 2 main categories: contractile dysfunction and myocyte loss. While both are considered important during the progression of DIC, myocyte loss might be more critical in the progression of this disease because it is irreversible and has a poor prognosis, sometimes proving fatal decades after its onset (1). Recently, RCD has been subdivided into various forms, such as pyroptosis, autophagy-dependent cell death, necroptosis, and ferroptosis (25), and it has been reported that several RCDs such as apoptosis, necroptosis, and autophagy are involved in DOX-induced myocyte loss (~~2, 4, 31–33~~2, 4, 32-34). Here, we highlight the significance of ferroptosis in DOX-induced cell death by analyzing major RCDs under DOX treatment in cultured cardiomyocytes and conclude that ferroptosis, along with apoptosis, is the major form of RCD in DOX-induced myocyte death both in vivo and in vitro. In this study, we also demonstrated that GPx4 downregulation and excessive LP production synergistically

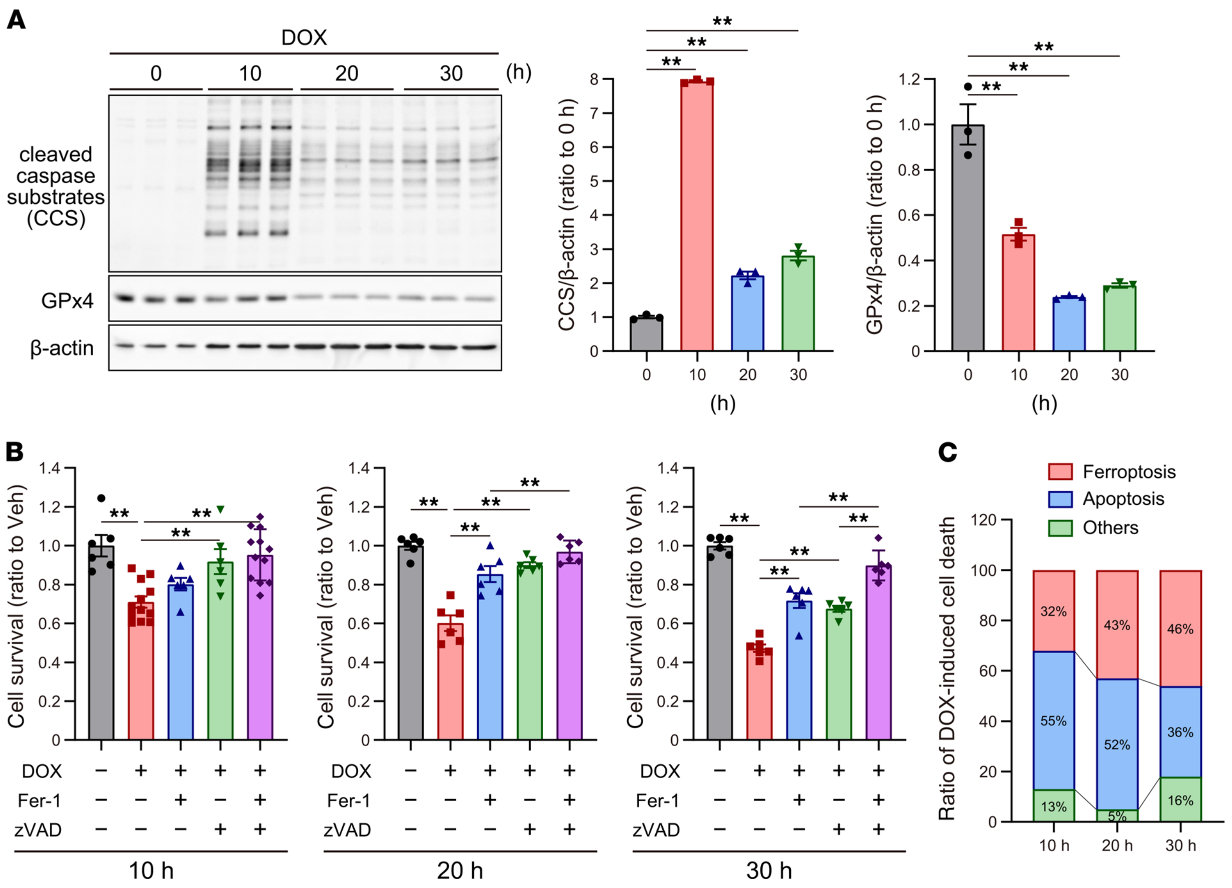


**Figure 9. Detailed analysis of cell death induced by DOX.** (**A**) Western blot of cleaved caspase substrate, cleaved caspase-3, and GPx4 in cultured cardiomyocytes, treated with DOX at 10, 20, and 30 hours after DOX treatment ($n = 3$). (**B**) Cell viability was assessed at 10, 20, and 30 hours after treatment with DOX, zVAD, and/or Fer-1 in cardiomyocytes ($n = 6$–$12$). (**C**) Details of cell death induced by DOX in cultured cardiomyocytes at each phase. Ferroptosis is defined as the percentage of cell death rescued by Fer-1 in DOX-induced cell death. Apoptosis is defined as the percentage of cell death rescued by zVAD in DOX-induced cell death. Others is defined as the percentage of cell death that was not rescued by a concomitant inhibition with Fer-1 and zVAD. Total percentage (100%) was divided into 3 categories (ferroptosis, apoptosis, and others) in accordance with their ratios. Detailed calculations are described in Supplemental Figure 9. Data are shown as the mean ± SEM. Statistical significance was determined using 1-way ANOVA with a post hoc Tukey's HSD test. *$P < 0.05$, **$P < 0.01$.

induced ferroptosis under DOX treatment, as either GPx4 overexpression or suppression of LPs by ~~chelating~~ reducing DOX-$Fe^{2+}$ effectively prevented DOX-induced ferroptosis. These essential requirements for the induction of ferroptosis under DOX treatment might be supported by the fact that the only heterodeletion of *Gpx4* is not sufficient to induce ferroptosis and cardiac dysfunction and that ~~chelation targeting $Fe^{2+}$~~ $Fe^{2+}$ reduction in mitochondria partially rescued the DOX-induced ferroptosis.

Our results also suggest that DOX-induced ferroptosis is triggered in mitochondria as DOX increased LPs in mitochondria, not in other organelles, and mitoGPx4-FLAG — mainly localized in mitochondria — fully suppressed the production of LPs and prevented DOX-induced ferroptosis. We previously reported that mitochondrial GPx4 could be imported into both the intermembrane space and cristae of mitochondria, while nonmitochondrial GPx4 was localized in the outer membrane and intermembrane space, but not in cristae of mitochondria (~~34~~35). In fact, the overexpression of mitochondrial GPx4, but not nonmitochondrial GPx4, suppressed mitochondrial oxidative stress (~~35~~36) and apoptosis mediated by the mitochondrial pathway in rat basophile leukemia cells (RBL2H3 cells) (~~36~~37), suggesting that nonmitochondrial GPx4 plays a limited role in mitochondria (~~37~~38). Furthermore, we also showed that the transgene of nonmitochondrial (cytosolic) *Gpx4* without MTS rescued cell death induced by the depletion of all isoforms of *Gpx4*, while that of mitochondrial *Gpx4* with MTS could not prevent it, indicating that nonmitochondrial (cytosolic) *Gpx4* was an essential isoform in GPx4 deletion–induced cell death (~~38~~39). However, the organelle that triggers ferroptosis remains controversial. Angeli et al. reported that ferroptosis inducers, such as erastin and RSL3, could induce ferroptosis without mitochondrial lipid peroxidation (12), and Kagan et al.

reported that 15-LOX oxidized phosphatidylethanolamine (PE) in the endoplasmic reticulum to generate PEOOH and enhanced lipid peroxidation by the Fenton reaction with cytosolic $Fe^{2+}$ in RSL3- or erastin-induced ferroptosis (~~39~~40). These results indicate that an iron-dependent lipid peroxidation was triggered in organelles excluding mitochondria. Therefore, we assumed that nonmitochondrial (cytosolic) GPx4 is a critical isoform, even in DOX-induced ferroptosis. However, an increase in LP levels in response to DOX treatment was confirmed only in the mitochondria in the heart tissues of DIC mice and cultured cardiomyocytes after DOX treatment. Furthermore, we unexpectedly found that nonmitochondrial GPx4 was localized not only in the cytosol, but also in the mitochondria, and that mitochondrial GPx4 (mainly localized in mitochondria) fully prevented DOX-induced ferroptosis. Taken together, mitochondria are major organelles for LP production in DOX-induced ferroptosis. This is also supported by evidence that the MFG ~~specifically chelates $Fe^{2+}$ in mitochondria~~ reduces $Fe^{2+}$ in mitochondria by converting $Fe^{2+}$ to $Fe^{3+}$ through its reaction (~~40~~31) and that it effectively suppressed mitochondrial LPs and prevented DOX-induced cell death. Recently, Gao et al. reported that cysteine-deprived ferroptosis is triggered by the oxidative stress derived from the electron transport chain (ETC) of mitochondria, with the evidence that ETC inhibitor, including rotenone and antimycin A, suppressed cysteine-deprived ferroptosis (41). We therefore examined the effect of rotenone and antimycin A on DOX-induced ferroptosis, but there was no protection against DOX-induced ferroptosis within the range without harmful effects in cultured cardiomyocytes (Supplemental Figure 10, A and B). These results indicated that DOX-induced ferroptosis is mechanistically different from cysteine-deprivation ferroptosis, as Gao et al. showed (41), though mitochondria are the common organelles in both types of ferroptosis. Additionally, we examined the role of glutathione-SH (GSH) in DOX-induced ferroptosis since GSH serves as a critical regulator of GPx4 activity as a cofactor. Interestingly, we found that the ratio of GSH/GSSG (glutathione-S-S-glutathione) was significantly reduced in cultured cardiomyocytes in response to DOX treatment (Supplemental Figure 11A). However, N-acetyl-L-cysteine (NAC) did not prevent DOX-induced ferroptosis in cultured cardiomyocytes, though it increased the GSH/GSSG ratio (Supplemental Figure 11, A and B). These results suggest that GSH starvation is not a limitation for GPx4 activity and that GPx4 expression, not GSH, is a key component of DOX-induced ferroptosis.

The efficacy of the iron ~~chelator~~ suppressor against $Fe^{2+}$, but not $Fe^{3+}$, in DOX-induced ferroptosis is one of the most important observations in this study. Ichikawa et al. reported that mitochondrial iron accumulation mediated the cardiotoxicity of DOX through DOX-induced downregulation of ABC protein-B8 (ABCB8), and Fang et al. recently showed that DOX upregulated heme oxygenase-1 (HO-1) in an NRF2-dependent manner, leading to heme degradation and accumulation of non-heme iron (5, 42). In fact, we also found that iron in mitochondria increased in the DIC model mice and in DOX-treated cardiomyocytes. However, there has been no evidence describing the effectiveness of DFO or DXZ, which are widely used against ferroptosis, on cell death induced by DOX, and the role of iron remains to be fully elucidated. Indeed, we showed that neither DFO nor DXZ prevented DOX-induced LPs or cell death in cultured cardiomyocytes. Given that MFG could ~~chelate not only free $Fe^{2+}$, but also DOX-$Fe^{2+}$~~ reduce not only free $Fe^{2+}$, but also DOX-$Fe^{2+}$, and that the offset of fluorescence intensity — provided by ~~the chelation against DOX-$Fe^{2+}$~~ reaction with DOX-$Fe^{2+}$ — was added in all groups under DOX treatment, both DFO and DXZ fully decreased free iron in the mitochondria (Figure 7C). Previous studies have shown that DOX can directly extract $Fe^{3+}$ from ferritin, resulting in the formation of a DOX-$Fe^{3+}$ complex, and that DOX-$Fe^{2+}$, which is the reduced form of DOX-$Fe^{3+}$, plays a central role in the DOX-induced Fenton reaction (26–30). Thus, it might be reasonable that iron chelators against free $Fe^{3+}$ are not effective in preventing DOX-induced LPs and ferroptosis, and that the ~~direct chelator against~~ direct suppressor against $Fe^{2+}$ is an effective inhibitor of the DOX-$Fe^{2+}$ complex. Although some studies demonstrated that iron chelation using DXZ ameliorated DIC in a mouse model (5, 42), we speculate that long-term treatment with DXZ in vivo might be effective for DIC, possibly irrespective of ferroptosis. We therefore suggest the possibility that ~~iron chelation against $Fe^{2+}$~~ $Fe^{2+}$ reduction, especially targeting mitochondria, would be more effective for directly preventing DOX-induced ferroptosis and DIC. As neither DFO nor DXZ are able to prevent DOX-induced ferroptosis, the ferroptosis described here might be different from that originally defined by Dixon et al. (9). However, we recognize DOX-induced ferroptosis as a subtype of ferroptosis because iron ~~chelators~~ suppressors targeting $Fe^{2+}$ effectively prevented it. On the other hand, the molecular mechanism by which iron accumulated in mitochondria remains controversial. We examined the expressions of HO-1 and ABCB8, as shown in previous studies (5, 42). However, we found no upregulation of HO-1 in our DIC model or cultured cardiomyocytes treated with DOX (Supplemental Figure 12, A–D). In addition, the role

of HO-1 in iron accumulation and DIC progression conflicts with the protective role of HO-1 in the DIC model reported by Hull et al. (24). On the other hand, ABCB8 was downregulated in DOX-treated cultured cardiomyocytes but not in our DIC model (Supplemental Figure 13, A and B). However, *Abcb8* silencing using siRNA in cultured cardiomyocytes did not sufficiently increase the iron content in mitochondria compared with that after DOX treatment, although *Abcb8* silencing reduced its transcriptional expression as effectively as DOX (Supplemental Figure 13, C and D). These results suggest that upregulation of HO-1 is not an essential component for iron accumulation in the mitochondria and DOX-induced ferroptosis and that downregulation of ABCB8 is not a sufficient mechanism for iron accumulation in the mitochondria after DOX treatment. Although we have deduced that DOX extracts iron from ferritin in the mitochondria, resulting in mitochondrial iron accumulation, further investigation is needed to clarify the pivotal mechanism of iron accumulation in the mitochondria after DOX treatment.

The higher susceptibility of cardiomyocytes to DOX-induced injury than that of other tumor cell types in DIC is interesting and has clinical implications. Of note, our examination using cancer cell lines demonstrated that GPx4 was downregulated by 2 μM DOX in cardiomyocytes but not in malignant cell lines, such as PC-3 and HeLa, at the same concentration (Supplemental Figure 14, A–C); ferroptosis was induced in cardiomyocytes but not in these malignant cell lines (Supplemental Figure 14, D–F), though DOX equally induced LPs in all cell types (Supplemental Figure 14, G–I). These results suggest that the downregulation of GPx4 is critical for DOX-induced ferroptosis and that differential behaviors of GPx4 expression in response to DOX might be responsible for the susceptibility of DOX-induced injuries through ferroptosis. In contrast, the mechanism by which GPx4 is downregulated in response to DOX treatment remains unknown. Our finding that reduction in the GPx4 level correlated with the reduction in the *Gpx4* mRNA level after DOX treatment suggests that GPx4 is downregulated mainly at the transcriptional level. Furthermore, if GPx4 is reduced at the translational level, overexpressed GPx4 would also be decreased after DOX treatment. The fact that overexpressed GPx4-FLAG was not reduced after DOX treatment in our experiments (Figure 6A) indicates that endogenous dysregulation of *Gpx4* at the transcriptional level is a major cause of GPx4 downregulation. The differential expression of topoisomerase isoforms, which are inhibited by DOX, might explain cardiotoxicities in some DIC models (3, 43, 44). Zhang et al. demonstrated that DNA double strand breaks due to DOX-dependent inhibition of Top2b lead to changes in gene expression, defective mitochondrial biogenesis, and ROS formation (3). Therefore, we also examined the role of Top2b in DOX-induced *Gpx4* downregulation. However, our experiment did not provide any evidence indicating that silencing *Top2b* with siRNA prevented DOX-dependent downregulation of *Gpx4* (Supplemental Figure 15, A and B). Although the detailed mechanisms of the different behaviors of *Gpx4* depending on cell types remain unknown, we suggest that ferroptosis, triggered by *Gpx4* downregulation and excessive LPs, may be the critical cell death pathway that underlies the susceptibility of cardiomyocytes to DOX-induced injuries.

On the contrary, we could not determine the significance of ferroptosis in the survival of DIC mice in this study because the severity of the DIC in the mice used in our experiments was lesser compared with that of DIC in the mice used in previous studies (3, 4), and no mice in our study died even at the 1-month period during which we monitored DIC progression. Hence, longer periods of monitoring might be needed to further investigate the role of ferroptosis in DIC in humans because it can often progress for long periods (1).

Finally, we discuss therapeutics that target ferroptosis in DIC. DXZ, which is the only drug approved by the US Food and Drug Administration for prevention of DOX-induced cardiotoxicity, might be an effective therapy, as shown clinically in meta-analysis (45). However, if we recognize ferroptosis as a therapeutic target in DIC, ~~iron chelators against~~ iron chelators or suppressors against $Fe^{2+}$ — especially in mitochondria — would be the ideal ~~chelators~~, as DXZ is not able to directly prevent DOX-induced ferroptosis. Given that DXZ is associated with some adverse effects, including secondary malignancies (46), the development of ideal iron ~~chelators~~ chelators or suppressors against $Fe^{2+}$ in mitochondria would be expected for a better cardioprotection against DOX-induced injuries in the future. Furthermore, lipophilic antioxidants against LPs such as Fer-1 used in this study are candidate therapeutic agents targeting ferroptosis. However, lipophilic antioxidants, even α-tocopherol (vitamin E), accumulate in the body and can sometimes induce adverse effects (47, 48). Hence, the development of an ideal therapeutic antioxidant, which would be lipophilic and nontoxic, will be required for any treatment modality that targets ferroptosis. Additionally, pretreatment with ferroptosis inhibitor is a key component for the effective prevention of DIC because our examination in cultured cardiomyocytes revealed that pretreatment is essential for the effective prevention of DOX-induced ferroptosis.

In summary, DOX downregulates GPx4 and induces ferroptosis triggered in mitochondria, resulting in DIC. Ferroptosis is the major form of cell death induced by DOX, and therapeutics targeting ferroptosis might be a novel preventive strategy for DIC.

## Methods

*Animal experiments*. Male C57BL/6J mice were housed in a temperature- and humidity-controlled room, fed a commercial diet (CRF-1; Oriental Yeast Co. Ltd.), and given free access to water. GPx4 Tg mice and GPx4 hetKO mice were produced as previously described (16, 17, 49). In these gene-manipulated mice, GPx4 was systemically overexpressed or absent, respectively. These strains were backcrossed with C57BL/6J mice in our laboratory. The DIC model was reproduced as previously reported, with some modification (24, 50). Briefly, DOX (6 mg/kg, body weight) was administered to mice via tail vein at days 0, 2, and 4. After euthanasia via pentobarbital overdose, the heart was excised and dissected into right and left ventricles, including the septum. Tibial length was measured after exposing the tibia by removing the skeletal muscle and soft tissue.

*Reagents*. DOX (D1515), Fer-1 (SML0583), necrostatin-1 (N9037), staurosporine (S4400), and antimycin A (A8674) were purchased from MilliporeSigma. zVAD (3118-v) was purchased from Peptide Institute. Rotenone (R0090) and NAC (A0905) were purchased from Tokyo Chemical Industry. DFO mesylate (ab120727) and DXZ hydrochloride (ab141109) were purchased from Abcam. MFG (M489) was purchased from Dojindo.

*Echocardiography*. Echocardiographic data were obtained through 2-dimensional targeted M-mode images, obtained in the short-axis view at the papillary muscle level using a Vevo 2100 ultrasonography system (Visual Sonics) under light anesthesia (1–2% isoflurane; ref. 51).

*Cell cultures*. Primary cultures of the isolated neonatal rat ventricular cardiomyocytes were prepared from the ventricles of neonatal Sprague Dawley rats as described previously, with some modifications (52). Neonatal rats were euthanized with an overdose of isoflurane, and the hearts were then rapidly excised. After digesting the myocardial tissues with trypsin (Thermo Fisher Scientific, 25300-062) and collagenase type 2 (Worthington Biochemical Corporation, LS004176), the cells were suspended in DMEM (Sigma-Aldrich, D5796) containing 10% FBS (HyClone Laboratories, SH30910.03) and 1% penicillin/streptomycin (P/S; Nacalai, 26253-84). Cells were plated twice in 100-mm culture dishes for 70 minutes each to reduce the number of nonmyocytes. Nonadherent cells were plated in culture dishes (Primaria) at $2.5 \times 10^5$/mL for each experiment as cultured cardiomyocytes. Adherent cells were passaged 2 times, and passaged cells (P2) were used as nonmyocytes, such as fibroblasts and endothelial cells. They were maintained at 37°C in humidified air with 5% $CO_2$. Rat H9c2 cells and human HeLa cells were maintained in DMEM containing 10% FBS and P/S at 37°C in humidified air with 5% $CO_2$. Human PC3 cells were maintained in RPMI 1640 medium (Thermo Fisher Scientific, 11875093) containing 10% FBS and P/S at 37°C in humidified air with 5% $CO_2$. DOX treatment started 24 hours after replacement with FBS-free medium, and Fer-1, zVAD, DFO, DXZ, MFG, Nec-1, rotenone, and antimycin A were added 30 minutes before DOX treatment.

*Adenovirus construction*. Adenoviruses harboring nonmitochondrial (cytosolic) and mitochondrial *Gpx4* were prepared as follows. Target sequences, which were cloned from a rat cDNA library, were inserted into pENTR-1A vector (cytoGPx4-Ad, attL1-SalI-kozak-GPx4 [cyto]-FLAG-SECIS-NotI-attL2; mitoGPx4-Ad, attL1-SalI-kozak-GPx4 [mito]-FLAG-SECIS-NotI-attL2). After confirmation of inserted sequences in the pENTR-1A vector, each target sequence was transferred to the adenovirus vector through an LR reaction. Recombinant sequences in the adenovirus vector were confirmed (Supplemental Figures 5 and 6).

*Transfection of siRNA and adenoviruses*. *Gpx4*, *Ripk3*, and *Top2b* knockdown was performed by siRNA transfection with Lipofectamine RNAiMAX (Thermo Fisher Scientific) according to the manufacturer's instructions. Briefly, 24 hours after seeding, the cardiomyocytes were transfected with siRNA and incubated for 48 hours. siRNAs used in this study were as follows: *Gpx4* (1 nM, s131172, Thermo Fisher Scientific), *Ripk3* (10 nM, s140858, Thermo Fisher Scientific), *Abcb8* (1 nM, s168896, Thermo Fisher Scientific), and *Top2b* (10 nM, s166822, Thermo Fisher Scientific). Cardiomyocytes were infected with adenovirus harboring cytoGPx4s (Ad-cytoGPx4) and mitoGPx4 (Ad-mitoGPx4) 48 hours after seeding. They were incubated for 24 hours; then, the medium was replaced with FBS-free medium. DOX treatment started 24 hours after replacement with FBS-free medium.

*Western blotting*. Western blotting was performed as previously described (53, 54). We used primary antibodies for GPx4 (Abcam, ab125066), acrolein (JaICA, MAR-020n), cleaved caspase substrate motif

(Cell Signaling Technology, 8698), COX IV (Cell Signaling Technology, 4844), FLAG (Cell Signaling Technology, 2368), cytochrome c (Cell Signaling Technology, 11940), RIP3K (Cell Signaling Technology, 15828), heme HO-1 (Enzo Life Sciences, ADI-SPA-895), Top2b (Abcam, ab125297), GAPDH (Santa Cruz Biotechnology Inc., sc-32233), and β-actin (Santa Cruz Biotechnology Inc., sc-47778).

*qPCR*. Total RNA extraction and quantitative PCR (qPCR) were performed as described previously, with some modifications (55). Briefly, total RNA was extracted using an RNeasy Mini Kit (Qiagen), RNA was converted to cDNA using ReverTra Ace qPCR RT Kit (TOYOBO), and the reactions were run in an Applied Biosystems QuantStudio3 for the THUNDERBIRD SYBR qPCR Mix. The forward (F) and reverse (R) primer sequences, respectively, were as follows: *Rps18* (mouse), F 5′-TTCTGGCCAACGGTCTAG-ACAAC-3′, R 5′-CCAGTGGTCTTGGTGTGCTGA-3′; total *Gpx4* (mouse), F 5′-CGCGATGATTGG-CGCT-3′, R 5′-CACACGAAACCCCTGTACTTATCC-3′; mitochondrial *Gpx4* (mouse), F 5′-AGCTG-GGGCCGTCTGAGCCG-3′, R 5′-ATGTCCTTGGCTGAGAATTCGT-3′; *heme oxygenase 1* (mouse), F 5′-TGCAGGTGATGCTGACAGAGG-3′, R 5′-GGGATGAGCTAGTGCTGATCTGG-3′; *Abcb8* (mouse), F 5′-GGGCCAGGTCATAGGTTTCATC-3′, R 5′-AACTCGTGGGCATTGGCTTC-3′; *Rps18* (rat), F 5′-AAGTTTCAGCACATCCTGCGAGTA-3′, R 5′-TTGGTGAGGTCAATGTCTGCTTTC-3′; total *Gpx4* (rat), F 5′-CCGGCTACAATGTCAGGTTT-3′, R 5′-ACGCAGCCGTTGTTATCAAT-3′; mitochondrial *Gpx4* (rat), F 5′-CGCTTATTGAAGCCAGCACT-3′, R 5′-TTATCCAGGCAAACCATGTG-3′; *heme oxygenase 1* (rat), F 5′-AGGTGCACATCCGTGCAGAG-3′, R 5′-CTTCCAGGGCCGTATAGATATGGTA-3′; *Abcb8* (rat), F 5′-GCAGCTAGTGAGCCGCTTGA-3′, R 5′-AGGTGTGACAACAGCCAGCAT-3′; *Top2b* (rat), F 5′-TCCAAGTCAAACAGTCGCTGCTA-3′, R 5′-CTTTGGAATGTTGCCGAGTTCA-3′.

*Histology*. For histological analysis, the apical myocardia were stored in 10% formalin for at least 4 days. Myocardia were embedded in paraffin, and 3-mm–sectioned samples were stained with Masson's trichrome (MT) reagent and TUNEL. TUNEL staining was performed to detect in situ DNA fragmentation using the in situ Apoptosis Detection Kit (Takara Bio). The in vivo specimens were counterstained by H&E.

*Isolation of cytosolic and mitochondrial fractions in vivo and in vitro*. Isolation of cytosolic and mitochondrial fractions was performed using heart tissues and cultured cardiomyocytes wherein the Mitochondria/Cytosol Fractionation Kit (ab65320, Abcam) was used for cytosol fractionation and the Mitochondria Isolation Kit (P507L, 101 Bio) for mitochondrial fractionation in accordance with the manufacturer's instructions.

*Measurements of MDA by TBARS assay*. MDA was measured using the thiobarbituric acid reactive substances (TBARS) assay kit (Cayman Chemical). Briefly, thiobarbituric acid was reacted with MDA in the samples at 100°C, and MDA-TBA adduct was fluorometrically measured (an excitation wavelength of 530 nm and an emission wavelength of 550 nm) using a Varioskan LUX Multimode Microplate Reader (Thermo Fisher Scientific).

*Measurements of mitochondrial lipid peroxidation using MitoPeDPP*. LPs in the mitochondrial inner membrane were measured using MitoPeDPP, which is a fluorescence probe that specifically reacts with LP in the mitochondrial inner membrane (Dojindo). Cultured cells were incubated in the presence of 0.5 μM MitoPeDPP solution for 30 minutes at 37°C. Following PBS washes, LPs in the mitochondrial inner membrane were fluorometrically measured (an excitation wavelength of 452 nm and an emission wavelength of 470 nm) using a Varioskan LUX Multimode Microplate Reader (Thermo Fisher Scientific) or observed using a fluorescence microscope (BZ-X800, Keyence).

*Measurement of lipid peroxidation by C11-BODIPY 581/591*. LPs were measured using C11-BODIPY 581/591 (Thermo Fisher Scientific). Briefly, after 30 hours of DOX treatment, the cells were incubated in 2 μM C11-BODIPY581/591 (Invitrogen) for 30 minutes at 37°C in the dark. They were then fluorometrically measured (an excitation wavelength of 581 nm and an emission wavelength of 591 nm) using a Varioskan LUX Multimode Microplate Reader (Thermo Fisher Scientific).

*Cell survival assay using Calcein AM*. To assess cell viability, the calcein-assay was performed using a Cell Counting Kit-F (Dojindo). Briefly, after washing with PBS, cultured cells were incubated in Calcein AM diluted solution in PBS (Calcein AM/PBS, 1:50) for 15 minutes at room temperature; then, cell viability was fluorometrically measured (an excitation wavelength of 490 nm and an emission wavelength of 520 nm) using a Varioskan LUX Multimode Microplate Reader (Thermo Fisher Scientific).

*IHC in vitro*. Cardiomyocytes were washed with PBS and incubated in culture medium containing 500 nM MitoTracker Red CMXRos (Invitrogen) for 30 minutes at 37°C. Following incubation, they were washed with PBS and fixed with 4% paraformaldehyde (PFA) for 10 minutes. After blocking with 1% BSA (MilliporeSigma) in PBS, they were incubated with FLAG antibody (Cell Signaling Technology, 2368P) at

4°C overnight. After washing with PBS, the FLAG antibody was labeled with a secondary antibody (Alexa Fluor 488 chicken anti–rabbit IgG, Invitrogen, A21441) for 1 hour. After washing, they were enclosed with mounting medium using DAPI (Vectashield, Vector Laboratories, H1200) and observed using a fluorescence microscope (BZ-X800, Keyence).

*IHEM in vitro*. The cultured cardiomyocytes on the gold disks were frozen in liquid propane at –175°C. Once the cultured cardiomyocytes were frozen, they were freeze substituted with 1% tannic acid in ethanol and 2% distilled water at –80°C for 24 hours. Afterward, they were transferred to –20°C for up to 3 hours and then warmed to 4°C for 4 hours. The samples were dehydrated through anhydrous ethanol 3 times for 30 minutes each and then infiltrated with a 50:50 mixture of ethanol and resin (LR white; London Resin Co. Ltd.) at 4°C for 1 hour. After this infiltration, 3 changes of 100% LR white at 4°C for 30 minutes each were performed. The samples were transferred to a fresh 100% resin and polymerized at 50°C overnight. The polymerized resins were ultrathin sectioned at 90 nm with a diamond knife using an ultramicrotome (Ultracut UCT; Leica), and the sections were placed on nickel grids. The grids were incubated with the FLAG antibody (Cell Signaling Technology, 2368P) in PBS containing 1% BSA at 4°C overnight, and then washed with PBS containing 1% BSA 3 times for 1 minute. They were subsequently incubated with a secondary antibody conjugated to 10 nm gold particles (goat anti–rabbit IgG) for 2 hours at room temperature; after washing with PBS, the grids were placed in 2% glutaraldehyde in 0.1M cacodylate buffer. Afterward, the grids were dried and then stained with 2% uranyl acetate for 15 minutes and with lead stain solution (MilliporeSigma) at room temperature for 3 minutes. The grids were observed using a transmission electron microscope (JEM-1400Plus; JEOL Ltd.) at an acceleration voltage of 100 kV. Digital images (3296 × 2472 pixels) were taken with a CCD camera (EM-14830RUBY2; JOEL Ltd.). Gold particles in the cytosol and mitochondria were quantified as the number of gold particles per each area of cytosol or mitochondria (per $\mu m^2$).

*Measurements of mitochondrial iron in vivo*. Mitochondria lysates were prepared from mitochondria derived from heart samples by centrifugation. Briefly, LV tissues were homogenized in HES buffer (10 mM Hepes-NaOH, 0.25M Sucrose, and 1 mM EDTA-2Na) and then centrifuged at 4000 *g* for 10 minutes at 4°C. Supernatants were collected, and mitochondria were isolated as pellets by centrifuging them at 13,000 *g* for 10 minutes at 4°C. Isolated mitochondria were lysed in radioimmunoprecipitation assay (RIPA) lysis buffer (25 mM Tris-HCl [pH 7.6], 150 mM NaCl, 1% NP-40, 1% sodium deoxycholate, 0.1% SDS). Mitochondrial iron quantification was conducted using Metalloassay Kit Ferrozine method (Metallogenics, FE31M). Mitochondrial lysates were mixed with 6M HCl until the pH reached 2 and were then spun down to obtain the supernatant. Samples were mixed with R-A buffer, and the baseline absorbance at 560 nm of the samples was determined (OD1) using Varioskan LUX Multimode Microplate Reader (Thermo Fisher Scientific). Then, R-R Chelate Color was added to the samples, and after 5 minutes of reaction, the absorbance at 560 nm was again determined (OD2). To measure iron concentration in the samples, the increase in absorbance, which is OD2−OD1, was calculated and compared with the standard curve.

*Measurements of mitochondrial iron in vitro*. Mitochondrial iron was measured using MFG, which is a fluorescence probe for the detection of $Fe^{2+}$ in mitochondria (Dojindo). After 30 hours of DOX treatment, the cells were incubated in 5 μM MFG solution for 30 minutes at 37°C in the dark. Following PBS washes, ammonium iron (II) sulfate (Nacalai, 19431-35) prepared with purified water was added to the cells (final concentration, 100 μM); then, mitochondrial iron was fluorometrically measured (an excitation wavelength of 505 nm and an emission wavelength of 535 nm) using a Varioskan LUX Multimode Microplate Reader (Thermo Fisher Scientific) or observed using a fluorescence microscope (BZ-X800, Keyence).

*Caspase-3/7 activity*. Caspase-3/7 activity was measured in accordance with the manufacturer's instructions. After DOX treatment, cultured cardiomyocytes, plated in a 96-well plate, were incubated with 2 mM CellEvent Caspase-3/7 Green Detection Reagent (C10423, Thermo Fisher Scientific) in PBS including 5% FBS for 30 minutes. Caspase-3/7 activity was measured by a Varioskan LUX Multimode Microplate Reader (at an excitation wavelength of 502 nm and an emission wavelength of 530 nm).

*Measurement of annexin V binding*. Annexin V binding was measured using annexin V, Alexa Fluor 488 conjugate (A13201, Thermo Fisher Scientific). After DOX treatment, cultured cardiomyocytes, plated in a 96-well plate, were incubated in 100 μL annexin V binding buffer (10 mM HEPES, 140 mM NaCl, and 2.5 mM $CaCl_2$, pH 7.4) plus 5 μL annexin V for 15 minutes. Annexin V binding was measured by a Varioskan LUX Multimode Microplate Reader (at an excitation wavelength of 495 nm and an emission wavelength of 519 nm).

*Flow cytometry analysis*. TUNEL staining in H9c2 was performed using the In Situ Apoptosis Detection Kit (Takara Bio), in accordance with the manufacturer's instructions. After permeabilizing the nuclear membrane with PBS containing 0.2% Triton-X, nuclei in H9c2 were counterstained with propidium iodide (PI). Cells were counted using BD FACSVerse (BD Biosciences), and the data were analyzed by FlowJo Software.

*Measurement of GSH and GSSG*. GSH and GSSG in cultured cardiomyocytes were measured using the GSSG/GSH Quantification Kit (Dojindo), in accordance with the manufacturer's instructions. Briefly, cultured cardiomyocyte samples were lysed in 10 mM HCl (80 μL). After 2 freeze-thaw cycles, 5% 5-sulfosalicylic acid (SSA, 20 μL) was added, and the samples were centrifuged at 8000 *g* for 10 minutes at 4°C. The supernatants were diluted using $H_2O$ to achieve a final SSA concentration of 0.5%. For GSSG measurement, a masking solution (2 μL) was first added to the diluted supernatant samples (100 μL). Thereafter, the samples (40 μL per well) were transferred to a 96-well plate, and buffer solution (60 μL per well) was added to each sample-containing well; the plate was then incubated at 37°C for 1 hour. The substrate solution (60 μL) and enzyme/coenzyme solution (60 μL) were then added to each well. After incubation at 37°C for 10 minutes, total glutathione and GSSG levels were measured using a Varioskan LUX Multimode Microplate Reader (Thermo Fisher Scientific) and a calibration curve.

*Statistics*. Data are shown as means ± SEM. To compare the groups, we used 2-tailed Student's *t* test or 1-way ANOVA, followed by Tukey's or Dunnett's post hoc test. $P < 0.05$ was considered significant. The JMP14 Pro software (SAS institute) was used for the statistical analysis.

*Study approval*. All procedures involving animals and animal care protocols were approved by the Committee on Ethics of Animal Experiments at the Kyushu University Graduate School of Medical and Pharmaceutical Sciences and performed in accordance with the Guidelines for Animal Experiments of Kyushu University (A30-145) and the *Guide for the Care and Use of Laboratory Animals* (National Academies Press, 2011).

## Author contributions

TT, MI, HD, SI, KO, and AI performed the experiments. TT, MI, TI, SM, TK, KY, HI, and HT interpreted data obtained from the experiments. TT, MI, and TI designed experimental protocols, wrote the manuscript, and prepared figures. MI and TI conceived the project. KY and HI critically advised and commented for the project. HT approved and supervised the project.

## Acknowledgments

We thank Midori Sato and Akiko Hanada for their excellent experimental techniques and technical support from the Research Support Center of the Graduate School of Medical Sciences at Kyushu University. This work was supported by JSPS KAKENHI grants (16H07049 and 18K15892 to MI, 17K09582 and and 20K08426 to TI, 17H03977 and 18K19405 to KY, and 15H04815 and 19H03655 to HT); research grants from the Takeda Science Foundation, the Uehara Memorial Foundation and the Japan Foundation for Applied Enzymology (Vascular Biology of Innovation; VBIC) (MI), YOKOYAMA Foundation for Clinical Pharmacology (YRY-1911 to MI), MSD Life Science Foundation, and Public Interest Incorporated Foundation (MI); AMED CREST grant no. JP19gm0910013 (KY and HI); and AMED grant no. 19ek0109339h0002 (HT).

Address correspondence to: Masataka Ikeda and Tomomi Ide, Department of Cardiovascular Medicine, Faculty of Medical Sciences, Kyushu University, 3-1-1 Maidashi, Higashi-ku, Fukuoka 812-8582, Japan. Phone: 81.92.642.5360; Email: ikeda-m@cardiol.med.kyushu-u.ac.jp (MI); tomomi_i@cardiol.med.kyushu-u.ac.jp (TI).

1. Felker GM, et al. Underlying causes and long-term survival in patients with initially unexplained cardiomyopathy. *N Engl J Med*. 2000;342(15):1077–1084.
2. Octavia Y, Tocchetti CG, Gabrielson KL, Janssens S, Crijns HJ, Moens AL. Doxorubicin-induced cardiomyopathy: from molecular mechanisms to therapeutic strategies. *J Mol Cell Cardiol*. 2012;52(6):1213–1225.
3. Zhang S, et al. Identification of the molecular basis of doxorubicin-induced cardiotoxicity. *Nat Med*. 2012;18(11):1639–1642.
4. Zhang T, et al. CaMKII is a RIP3 substrate mediating ischemia- and oxidative stress-induced myocardial necroptosis. *Nat Med*. 2016;22(2):175–182.
5. Ichikawa Y, et al. Cardiotoxicity of doxorubicin is mediated through mitochondrial iron accumulation. *J Clin Invest*. 2014;124(2):617–630.

6. Lebrecht D, Setzer B, Ketelsen UP, Haberstroh J, Walker UA. Time-dependent and tissue-specific accumulation of mtDNA and respiratory chain defects in chronic doxorubicin cardiomyopathy. *Circulation*. 2003;108(19):2423–2429.
7. Xiong Y, Liu X, Lee CP, Chua BH, Ho YS. Attenuation of doxorubicin-induced contractile and mitochondrial dysfunction in mouse heart by cellular glutathione peroxidase. *Free Radic Biol Med*. 2006;41(1):46–55.
8. Yen HC, Oberley TD, Vichitbandha S, Ho YS, St Clair DK. The protective role of manganese superoxide dismutase against adriamycin-induced acute cardiac toxicity in transgenic mice. *J Clin Invest*. 1996;98(5):1253–1260.
9. Dixon SJ, et al. Ferroptosis: an iron-dependent form of nonapoptotic cell death. *Cell*. 2012;149(5):1060–1072.
10. Stockwell BR, et al. Ferroptosis: A Regulated Cell Death Nexus Linking Metabolism, Redox Biology, and Disease. *Cell*. 2017;171(2):273–285.
11. Zilka O, et al. On the Mechanism of Cytoprotection by Ferrostatin-1 and Liproxstatin-1 and the Role of Lipid Peroxidation in Ferroptotic Cell Death. *ACS Cent Sci*. 2017;3(3):232–243.
12. Angeli JP, et al. Inactivation of the ferroptosis regulator Gpx4 triggers acute renal failure in mice. *Nat Cell Biol*. 2014;16(12):1180–1191.
13. Sneddon AA, et al. Regulation of selenoprotein GPx4 expression and activity in human endothelial cells by fatty acids, cytokines and antioxidants. *Atherosclerosis*. 2003;171(1):57–65.
14. Imai H, Matsuoka M, Kumagai T, Sakamoto T, Koumura T. Lipid Peroxidation-Dependent Cell Death Regulated by GPx4 and Ferroptosis. *Curr Top Microbiol Immunol*. 2017;403:143–170.
15. Wilting R, Schorling S, Persson BC, Böck A. Selenoprotein synthesis in archaea: identification of an mRNA element of Methanococcus jannaschii probably directing selenocysteine insertion. *J Mol Biol*. 1997;266(4):637–641.
16. Imai H, et al. Early embryonic lethality caused by targeted disruption of the mouse PHGPx gene. *Biochem Biophys Res Commun*. 2003;305(2):278–286.
17. Imai H, et al. Depletion of selenoprotein GPx4 in spermatocytes causes male infertility in mice. *J Biol Chem*. 2009;284(47):32522–32532.
18. Ueta T, et al. Glutathione peroxidase 4 is required for maturation of photoreceptor cells. *J Biol Chem*. 2012;287(10):7675–7682.
19. Chen L, Hambright WS, Na R, Ran Q. Ablation of the Ferroptosis Inhibitor Glutathione Peroxidase 4 in Neurons Results in Rapid Motor Neuron Degeneration and Paralysis. *J Biol Chem*. 2015;290(47):28097–28106.
20. Seiler A, et al. Glutathione peroxidase 4 senses and translates oxidative stress into 12/15-lipoxygenase dependent- and AIF-mediated cell death. *Cell Metab*. 2008;8(3):237–248.
21. Angeli JPF, Shah R, Pratt DA, Conrad M. Ferroptosis Inhibition: Mechanisms and Opportunities. *Trends Pharmacol Sci*. 2017;38(5):489–498.
22. Lebrecht D, et al. The 6-maleimidocaproyl hydrazone derivative of doxorubicin (DOXO-EMCH) is superior to free doxorubicin with respect to cardiotoxicity and mitochondrial damage. *Int J Cancer*. 2007;120(4):927–934.
23. Martinel Lamas DJ, et al. Selective cytoprotective effect of histamine on doxorubicin-induced hepatic and cardiac toxicity in animal models. *Cell Death Discov*. 2015;1:15059.
24. Hull TD, et al. Heme oxygenase-1 regulates mitochondrial quality control in the heart. *JCI Insight*. 2016;1(2):e85817.
25. Galluzzi L, et al. Molecular mechanisms of cell death: recommendations of the Nomenclature Committee on Cell Death 2018. *Cell Death Differ*. 2018;25(3):486–541.
26. Sugioka K, Nakano M. Mechanism of phospholipid peroxidation induced by ferric ion-ADP-adriamycin-co-ordination complex. *Biochim Biophys Acta*. 1982;713(2):333–343.
27. Zweier JL. Reduction of O2 by iron-adriamycin. *J Biol Chem*. 1984;259(10):6056–6058.
28. Miura T, Muraoka S, Ogiso T. Effect of ascorbate on adriamycin-Fe(3+)-induced lipid peroxidation and DNA damage. *Pharmacol Toxicol*. 1994;74(2):89–94.
29. Miura T, Muraoka S, Ogiso T. Adriamycin-induced lipid peroxidation of erythrocyte membranes in the presence of ferritin and the inhibitory effect of ceruloplasmin. *Biol Pharm Bull*. 1993;16(7):664–667.
30. Miura T, Muraoka S, Ogiso T. Lipid peroxidation of rat erythrocyte membrane induced by adriamycin-Fe3+. *Pharmacol Toxicol*. 1991;69(4):296–300.

~~40~~31. Hirayama T, Kadota S, Niwa M, Nagasawa H. A mitochondria-targeted fluorescent probe for selective detection of mitochondrial labile Fe(ii). *Metallomics*. 2018;10(6):794–801.

~~31~~32. Zhu W, et al. Acute doxorubicin cardiotoxicity is associated with p53-induced inhibition of the mammalian target of rapamycin pathway. *Circulation*. 2009;119(1):99–106.

~~32~~33. Shi J, Abdelwahid E, Wei L. Apoptosis in Anthracycline Cardiomyopathy. *Curr Pediatr Rev*. 2011;7(4):329–336.

~~33~~34. Dirks-Naylor AJ. The role of autophagy in doxorubicin-induced cardiotoxicity. *Life Sci*. 2013;93(24):913–916.

~~34~~35. Arai M, et al. Import into mitochondria of phospholipid hydroperoxide glutathione peroxidase requires a leader sequence. *Biochem Biophys Res Commun*. 1996;227(2):433–439.

~~35~~36. Arai M, et al. Mitochondrial phospholipid hydroperoxide glutathione peroxidase plays a major role in preventing oxidative injury to cells. *J Biol Chem*. 1999;274(8):4924–4933.

~~36~~37. Nomura K, Imai H, Koumura T, Arai M, Nakagawa Y. Mitochondrial phospholipid hydroperoxide glutathione peroxidase suppresses apoptosis mediated by a mitochondrial death pathway. *J Biol Chem*. 1999;274(41):29294–29302.

~~37~~38. Imai H, Nakagawa Y. Biological significance of phospholipid hydroperoxide glutathione peroxidase (PHGPx, GPx4) in mammalian cells. *Free Radic Biol Med*. 2003;34(2):145–169.

~~38~~39. Imai H. New Strategy of Functional Analysis of PHGPx Knockout Mice Model Using Transgenic Rescue Method and Cre-LoxP System. *J Clin Biochem Nutr*. 2010;46(1):1–13.

~~39~~40. Kagan VE, et al. Oxidized arachidonic and adrenic PEs navigate cells to ferroptosis. *Nat Chem Biol*. 2017;13(1):81–90.

41. Gao M, et al. Role of Mitochondria in Ferroptosis. *Mol Cell*. 2019;73(2):354–363.e3.
42. Fang X, et al. Ferroptosis as a target for protection against cardiomyopathy. *Proc Natl Acad Sci USA*. 2019;116(7):2672–2680.
43. Capranico G, Tinelli S, Austin CA, Fisher ML, Zunino F. Different patterns of gene expression of topoisomerase II isoforms in differentiated tissues during murine development. *Biochim Biophys Acta*. 1992;1132(1):43–48.
44. Khiati S, et al. Mitochondrial topoisomerase I (top1mt) is a novel limiting factor of doxorubicin cardiotoxicity. *Clin Cancer Res*.

2014;20(18):4873–4881.
45. Abdel-Qadir H, et al. Interventions for preventing cardiomyopathy due to anthracyclines: a Bayesian network meta-analysis. *Ann Oncol*. 2017;28(3):628–633.
46. Tebbi CK, et al. Dexrazoxane-associated risk for acute myeloid leukemia/myelodysplastic syndrome and other secondary malignancies in pediatric Hodgkin's disease. *J Clin Oncol*. 2007;25(5):493–500.
47. Miller ER, Pastor-Barriuso R, Dalal D, Riemersma RA, Appel LJ, Guallar E. Meta-analysis: high-dosage vitamin E supplementation may increase all-cause mortality. *Ann Intern Med*. 2005;142(1):37–46.
48. Bjelakovic G, Nikolova D, Gluud LL, Simonetti RG, Gluud C. Antioxidant supplements for prevention of mortality in healthy participants and patients with various diseases. *Cochrane Database Syst Rev*. 2012;(3):CD007176.
49. Yoshida M, et al. Involvement of cigarette smoke-induced epithelial cell ferroptosis in COPD pathogenesis. *Nat Commun*. 2019;10(1):3145.
50. Ikeda S, et al. Blockade of L-type $Ca^{2+}$ channel attenuates doxorubicin-induced cardiomyopathy via suppression of CaMKII-NF-κB pathway. *Sci Rep*. 2019;9(1):9850.
51. Ikeda M, et al. The Akt-mTOR axis is a pivotal regulator of eccentric hypertrophy during volume overload. *Sci Rep*. 2015;5:15881.
52. Fujino T, et al. Recombinant mitochondrial transcription factor A protein inhibits nuclear factor of activated T cells signaling and attenuates pathological hypertrophy of cardiac myocytes. *Mitochondrion*. 2012;12(4):449–458.
53. Inoue T, et al. Twinkle overexpression prevents cardiac rupture after myocardial infarction by alleviating impaired mitochondrial biogenesis. *Am J Physiol Heart Circ Physiol*. 2016;311(3):H509–H519.
54. Ikeda M, et al. Overexpression of TFAM or Twinkle increases mtDNA copy number and facilitates cardioprotection associated with limited mitochondrial oxidative stress. *PLoS One*. 2015;10(3):e0119687.
55. Arai S, et al. Functional loss of DHRS7C induces intracellular Ca2+ overload and myotube enlargement in C2C12 cells via calpain activation. *Am J Physiol, Cell Physiol*. 2017;312(1):C29–C39.
